# Estimating maize canopy water content using UAV-based multispectral–thermal infrared imagery and canopy signal distributional features

**DOI:** 10.3389/fpls.2026.1868370

**Published:** 2026-07-09

**Authors:** Wenwen Li, Xuyu Feng, Taisheng Du, Shaozhong Kang, Haiyang Zhang, Ling Tong

**Affiliations:** 1 State Key Laboratory of Efficient Utilization of Agricultural Water Resources, Beijing, China; 2 National Field Observation and Research Station (Gansu Wuwei) for Efficient Water Utilization in Oasis Agriculture, Wuwei, China; 3 Center for Agricultural Water Research in China, China Agricultural University, Beijing, China

**Keywords:** canopy water content, distributional features, maize, multispectral–thermal infrared imagery, spatial aggregation, UAV remote sensing

## Abstract

**Introduction:**

Canopy water content (CWC) is an important indicator of crop water status **and** supports precision irrigation decision-making. Plot-level CWC estimation using UAV imagery often relies on canopy mean features, whereas the role of within-plot canopy-signal distributional information remains insufficiently examined.

**Methods:**

In this study, spring maize at the Shiyanghe site was monitored using UAV-based multispectral and thermal infrared imagery. Mean, percentile, and dispersion features were extracted from effective canopy pixels within each plot. RFECV feature selection, 50 repeated random train–test splits, paired statistical tests, simulated spatial aggregation, and four regression models were used to evaluate the stage- and scale-dependent contribution of these features.

**Results and discussion:**

Water stress affected both overall spectral–thermal responses and within-plot signal distributions. Before tasseling, percentile and dispersion features were frequently selected and provided complementary information, especially for tree-based models and finer aggregation scales. After tasseling, mean features generally showed more stable performance, although some distributional features still contained CWC-related information. The supplementary Xinxiang site-internal analysis suggested that, under weak water-gradient and small-sample conditions, distributional features may be frequently selected but may not consistently improve prediction accuracy. Overall, the contribution of distributional features was growth-stage-, scale-, and model-dependent.

## 1 Introduction

Precision agriculture aims to identify and manage within-field spatial variability, enabling site-specific management according to crop growth status and resource conditions and thereby improving resource-use efficiency and agricultural sustainability (Mulla, 2013). Among various management practices, irrigation management depends particularly on timely and reliable diagnosis of crop water status, because irrigation timing and amount are largely determined by the accurate identification of crop water deficits across space and time. This requirement has become increasingly important under the combined pressures of growing water-resource constraints and the need to improve agricultural production efficiency. Therefore, identifying crop water status is a critical prerequisite for improving crop water productivity and supporting precision irrigation management (Jones et al., 2009; Dong et al., 2024). However, conventional diagnosis of crop water status still relies heavily on point-based measurements, such as soil moisture or physiological indicators. Although these methods are physiologically relevant, they are difficult to extrapolate to field-scale spatial management because they cannot adequately capture the spatial heterogeneity of crop responses within plots or fields (Jones, 2004; Ma et al., 2026).

Among the various indicators used to characterize crop water status, canopy water content (CWC) provides an integrated measure of crop water status and its response to water deficit at the canopy level. It is an important phenotypic indicator linking crop physiological status with changes in canopy structure. For field crops such as maize, which are relatively sensitive to water deficit, changes in CWC are not only associated with leaf water loss but are also closely related to stomatal regulation, transpiration processes, canopy structural changes, and overall crop growth status. Previous studies have shown that crop water content can be estimated using optical signals related to water absorption and canopy reflectance, leading to the development of a range of spectral indices and estimation methods across leaf, canopy, and whole-season scales (Ceccato et al., 2002; Clevers et al., 2008, 2010; Zhang et al., 2019a; Zhou et al., 2022).

In recent years, unmanned aerial vehicles (UAVs) have been widely used for field-scale monitoring of crop water stress because they enable flexible observation timing and efficient integration of multiple sensors (Maes and Steppe, 2019; Ahmad et al., 2021; Ndlovu et al., 2024). Machine learning methods applied to UAV remote sensing data can further improve the estimation of crop water-related traits, including maize leaf water content and canopy water status (Ndlovu et al., 2021; Meiyan et al., 2022). Multispectral imagery mainly captures canopy reflectance, pigment status, and structural changes, whereas thermal infrared imagery responds more directly to transpiration suppression and changes in canopy energy balance induced by water deficit (Berni et al., 2009; Costa et al., 2013). Several studies have integrated multispectral, thermal, hyperspectral, structural, meteorological and *in-situ* information with machine learning and deep learning methods to improve crop water-status and canopy-trait estimation (Taghvaeian et al., 2012; DeJonge et al., 2015; Zhang et al., 2019b; Leinonen and Jones, 2004; Moller et al., 2006; Meron et al., 2013). Despite these advances, many UAV-based crop water-status studies still rely mainly on plot-level mean features for model development. Mean-based features are useful and interpretable, but they may smooth within-plot variation in canopy reflectance, temperature, and water-stress-related signals. In high-resolution UAV imagery, water deficit may appear not only as a shift in the average canopy condition but also as changes in the pixel-value distribution, including lower or upper tails, dispersion, and localized anomalous patches. Therefore, image-derived canopy-signal distributional features may provide complementary information for plot-level CWC estimation.

Thermal infrared studies provide important evidence for this perspective. For example, canopy-temperature distribution metrics, such as standard deviation and range, have been shown to contain useful stress-related information beyond mean canopy temperature (Han et al., 2016; Luan et al., 2021). Meanwhile, water-stress indices based on mean canopy temperature can be affected by environmental conditions and baseline fluctuations (DeJonge et al., 2015; Zhang et al., 2023). These findings suggest that relying solely on mean thermal features may be insufficient for stable characterization of crop water status, and that thermal distributional features can provide complementary information.

Similar perspectives have also emerged in the estimation of other phenotypic traits. In addition to mean values, pixel-value distributions can be described using statistical or structural features such as percentiles, variance, range, coefficient of variation, and texture. These features can capture how canopy responses are organized within the canopy. Classic image analysis studies have long shown that spatial arrangement and distribution patterns can improve target recognition and characterization (Haralick et al., 1973). More recent UAV studies have also shown that integrating statistical, spectral, texture, multispectral, and thermal features can improve the estimation of crop growth parameters and canopy traits (Zhang et al., 2022; Sun et al., 2023; Yu et al., 2024; Li et al., 2026). However, these studies have mainly focused on LAI estimation, growth-status identification, or other canopy traits, with less attention paid to CWC as a direct indicator of canopy water status. Therefore, whether image-derived distributional features provide additional predictive information for plot-level maize CWC estimation remains unclear.

Another issue that should not be overlooked is the scale dependence of these statistical features. Because they are derived from pixel-value distributions, both their numerical values and physical interpretations are closely related to spatial resolution and the degree of pixel aggregation. Scale effects have long been a central issue in remote sensing, as changes in resolution can alter local variance, texture responses, and the proportion of mixed pixels, thereby changing the relationships between image features and biophysical variables. In thermal infrared remote sensing, Hou et al. (2021) reported that ground-based and UAV thermal infrared imagery can differ in crop transpiration estimation because of scale effects. However, in plot-scale maize CWC estimation, it remains unclear how UAV-derived distributional features respond to simulated spatial aggregation and which features remain relatively stable under resolution degradation.

**Figure 1:**
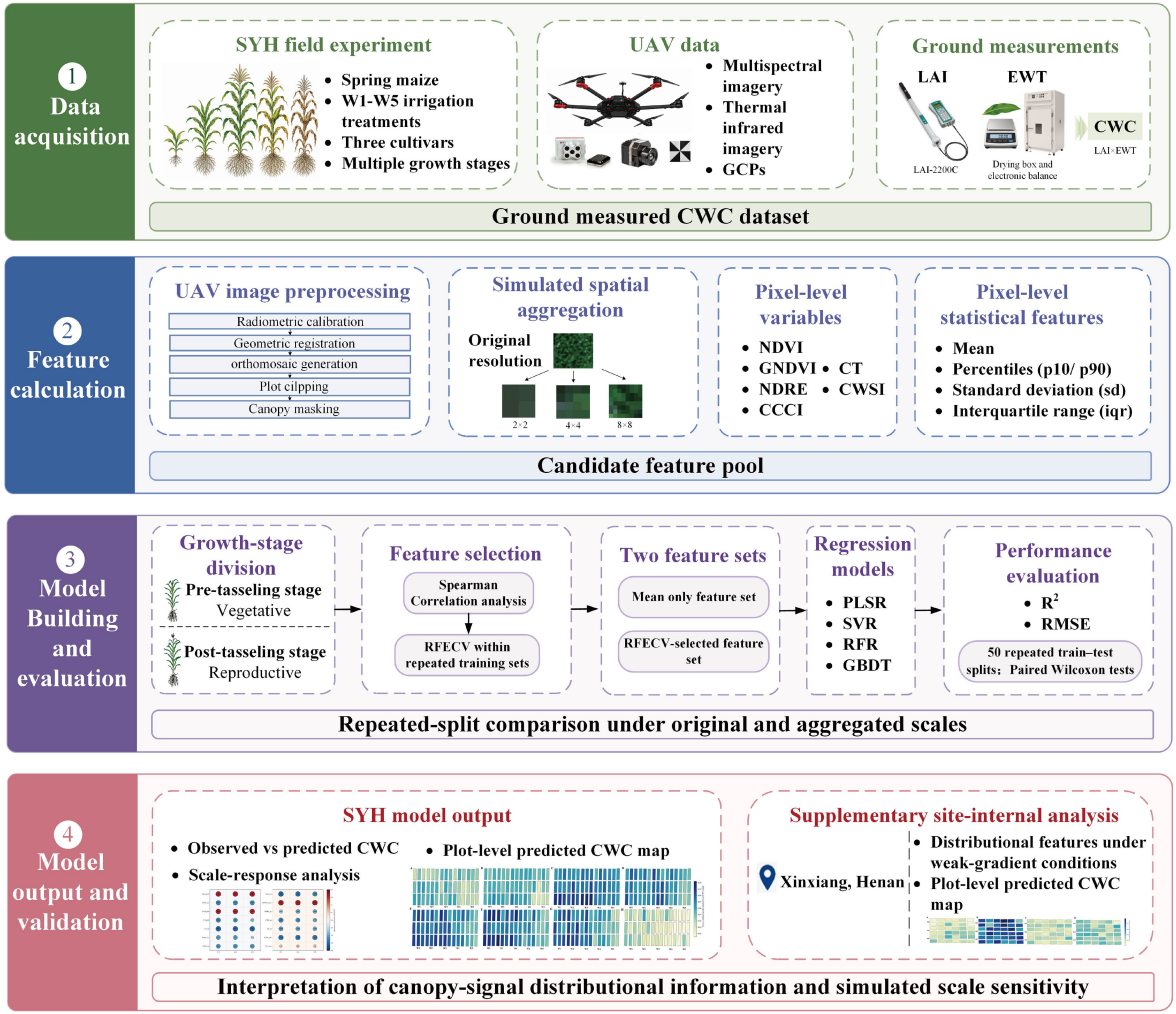
Overall flow chart.

Based on these considerations, this study used UAV-based multispectral and thermal infrared imagery to estimate plot-level maize CWC. Specifically, we examined the role of image-derived pixel-distribution-based features in characterizing within-plot canopy-signal heterogeneity and improving plot-level CWC estimation, compared the contributions of mean features and distributional statistical features, and evaluated the responses of these features and model performance under simulated spatial aggregation. The overall workflow of this study is shown in **Figure 1**.

## 2 Materials and methods

### 2.1 Study area and experimental design

#### 2.1.1 Study area

**Figure 2:**
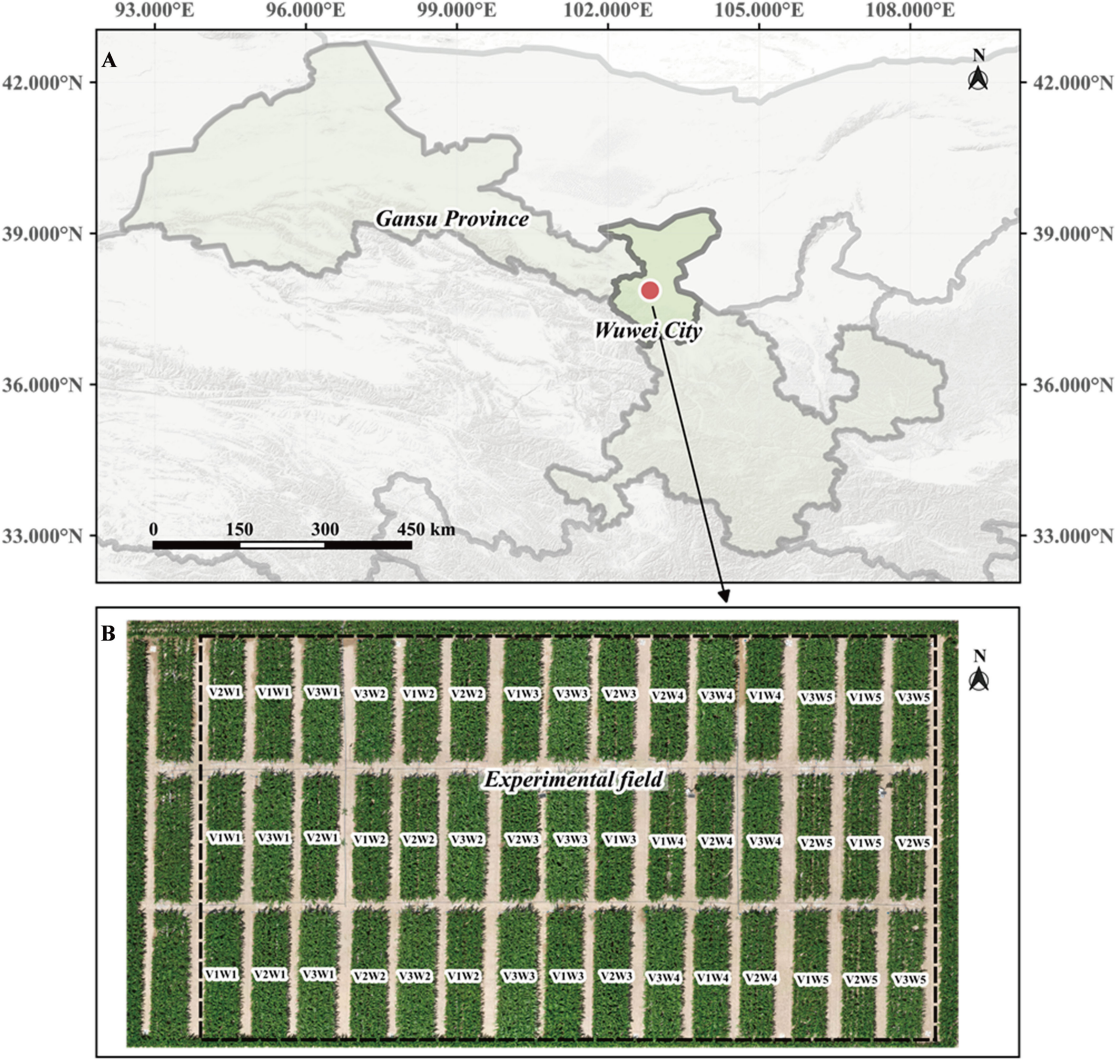
Overview of the study area and experimental design: **(A) **Gographical location of the Wuwei experimental site in Gansu Province, China; **(B)** layout of the experimental plots.

**Figure 3:**
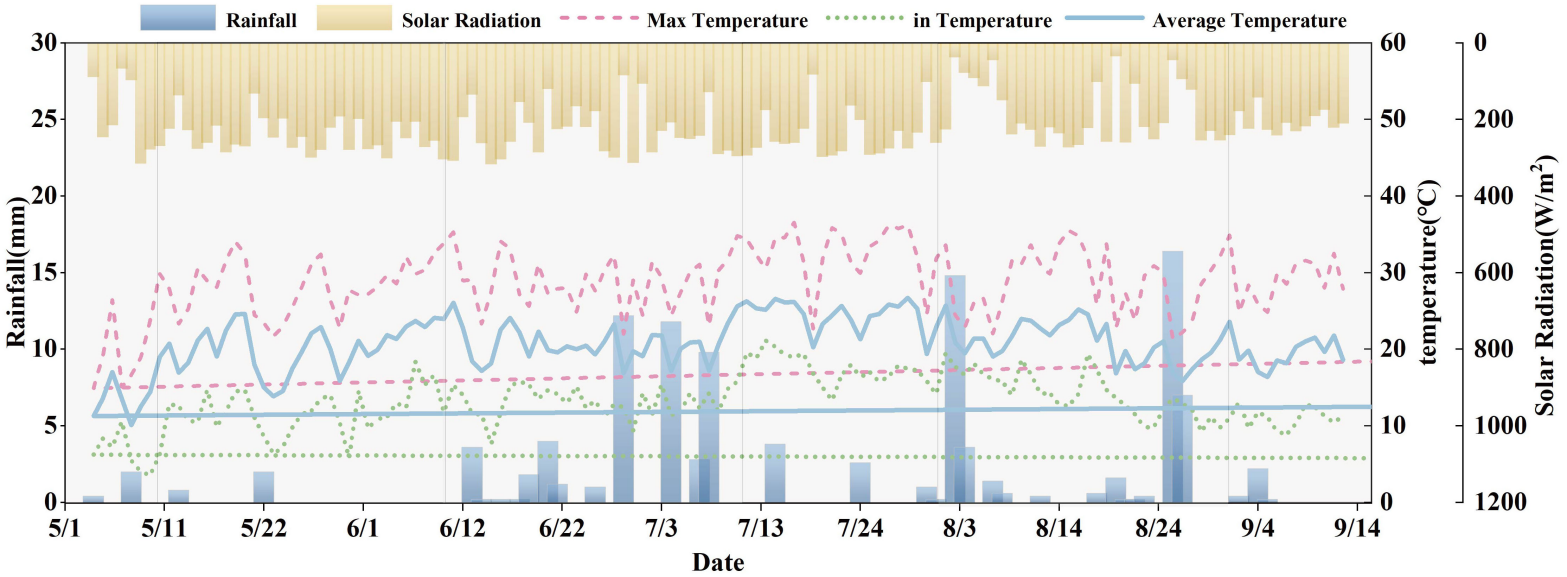
Meteorological conditions during the 2025 maize growing season.

The experiment was conducted from April to October 2025 at the National Field Scientific Observation and Research Station for Efficient Water Use in Oasis Agriculture, Wuwei, Gansu Province. The station is located in Wangjingzhai Village, Jinhe Town, Liangzhou District, Wuwei City, Gansu Province, China (37°52′20″N, 102°50′50″E; 1581 m a.s.l.; **Figure 2**). The region has a mean annual precipitation of 164.4 mm, annual evaporation exceeding 2000 mm, and a groundwater table deeper than 25 m. It is also characterized by abundant light and heat resources, with more than 3000 h of annual sunshine, a frost-free period of 150 days, and an accumulated temperature above 10 °C of more than 3256 °C. The soil texture in the experimental area is sandy loam. Within the 0–1 m soil layer, the mean soil bulk density and field capacity are 1.53 g/cm^3^ and 0.21 cm^3^/cm^3^, respectively. Meteorological data during the 2025 maize growing season were continuously recorded by a HOBO automatic weather station located near the experimental area. The recorded variables included air temperature, relative humidity, and precipitation. Based on these observations, daily air temperature and precipitation dynamics during the growing season were summarized. These data were aggregated to a daily scale to describe the environmental background during the experiment and the meteorological differences among observation dates (**Figure 3**).

#### 2.1.2 Experimental design

The spring maize cultivars used in this field experiment were XY 335 (V1), XY 1225 (V2), and ZD 958 (V3). Maize was planted in a one-film, three-row pattern, with rows oriented north–south. A mulched drip irrigation system was used because of its high water-saving efficiency. The plastic film was a locally used transparent plastic film with a thickness of 0.01 mm. Two drip irrigation tapes were installed under each film strip to irrigate three crop rows. The emitter flow rate of the drip tapes was 2.0 L h^-1^, and the irrigation amount in each experimental plot was controlled using a water meter. The average row spacing was 60 cm, and the plant spacing was 20 cm.

Five irrigation treatments were established: W1, mild regulated deficit irrigation at 65% ET_c_; W2, moderate regulated deficit irrigation at 40% ET_c_; W3, full irrigation; W4, mild deficit irrigation throughout the whole growing season at 65% ET_c_; and W5, moderate deficit irrigation throughout the whole growing season at 40% ET_c_. The irrigation amount was determined by calculating reference crop evapotranspiration (ET_0_) using the Penman–Monteith equation and then combining ET_0_ with crop coefficients for different maize growth stages and effective precipitation to obtain the actual irrigation quota for each treatment. The irrigation frequency was kept consistent with local practice. Irrigation was withheld during the early seedling stage for seedling hardening, and no irrigation treatment was applied during this period. The main irrigation events were conducted on May 4, June 11–13, June 27–30, July 17, July 28, and August 16, 2025. The first irrigation event on May 4 was applied uniformly to all treatments before the water-deficit treatments were imposed. Thereafter, treatment-specific irrigation amounts were applied according to the regulated-deficit and whole-season deficit irrigation schemes. The total irrigation amounts during the growing season were 234.39, 207.69, 271.76, 192.40, and 135.71 mm for W1, W2, W3, W4, and W5, respectively. For the regulated deficit irrigation treatments, water deficit was imposed during the late seedling, jointing, and late grain-filling stages. The field experiment followed a split-plot design with three replicates, in which irrigation treatment was the main-plot factor and maize cultivar was the subplot factor. The five irrigation treatments and three cultivars formed 15 irrigation–cultivar combinations, resulting in 45 plots in total. Each plot measured 3.5 m × 12 m. Detailed information on the experimental design, observation dates, and final plot-level CWC sample sizes used for model development is provided in **Supplementary Table 1**.

All treatments received the same amounts of basal fertilizer and topdressing fertilizer. In local maize production, herbicides and insecticides are usually applied before film mulching to prevent weed growth and pest occurrence. Therefore, the same herbicides and insecticides as those used in local practice were applied in this experiment. Other field management practices were also kept consistent with local maize production.

### 2.2 Data acquisition and processing

#### 2.2.1 Ground measurements

Ground sampling was conducted at different maize growth stages to obtain measured plot-levels canopy water content (CWC). In each plot, representative canopy areas were selected for LAI measurements and destructive leaf sampling, while avoiding border rows. Leaf area index (LAI) was measured using an LAI-2200C plant canopy analyzer. Five LAI measurement points were arranged within each plot, including one point near the plot center and four points distributed in non-border rows to represent within-plot canopy variation. Plot-level LAI was calculated as the mean of these measurements.

For destructive leaf sampling, two representative plants were selected from each plot on each sampling date, and one representative functional leaf was collected from each plant. Thus, two leaves were sampled per plot for EWT determination. Leaf equivalent water thickness (EWT) was determined from leaf fresh weight, leaf area, and dry weight after oven drying. Before tasseling, the third leaf from the top was selected as the representative functional leaf, whereas after tasseling, the ear leaf was selected. This leaf-position strategy was adopted according to maize phenological development, because the dominant functional leaf position changes from the upper fully expanded leaves before tasseling to the ear leaf after tasseling. After sampling, leaf fresh weight was measured immediately, and leaf area was determined before oven drying. The samples were then oven-dried to constant weight, and leaf dry weight was measured.

*LAI* represents the total leaf area per unit ground area, whereas *EWT* represents the leaf water mass per unit leaf area. Based on their multiplicative relationship, canopy water content (CWC) was calculated using Equation 1. *EWT* was derived from leaf fresh weight, dry weight, and leaf area, as shown in Equation 2, where *FW* is leaf fresh weight, *DW* is leaf dry weight, and *A* is leaf area. Finally, *LAI*, *EWT*, and *CWC* were summarized at the plot level. Therefore, the subsequent modeling target represented plot-level ground-measured CWC rather than pixel-level CWC.

(1) CWC=LAI×EWT

(2) $$\mathrm{EWT}=\frac{\mathrm{FW}-\mathrm{DW}}{A}$$

Because CWC was derived from LAI and EWT, measurement uncertainties in both variables may propagate to the final CWC values. LAI may be influenced by within-plot canopy heterogeneity and the representativeness of the five measurement points, whereas EWT may be affected by leaf-position selection, leaf-area measurement, fresh- and dry-weight measurements, and possible water loss before weighing. To reduce these potential effects, LAI measurement and leaf sampling were conducted within non-border rows, and leaf fresh weight was measured immediately after sampling. To account for potential stage-related differences associated with changes in representative leaf position, leaf sampling followed maize phenology, and model development was conducted separately for pre- and post-tasseling stages. The final plot-level CWC sample sizes used for model development are summarized in **Supplementary Table 1**.

#### 2.2.2 UAV image acquisition and preprocessing

Maize canopy images were acquired using a DJI Matrice 600 UAV platform. A MicaSense RedEdge multispectral camera was used to acquire five-band multispectral canopy imagery, and a radiometrically calibrated FLIR Vue Pro R thermal infrared camera was used to acquire thermal infrared imagery.

To reduce the effects of changes in illumination and meteorological conditions, all UAV flights were conducted under clear and stable illumination conditions and were completed on the same days as the ground measurements. The flight altitude was set to 50 m for all flights. UAV image acquisition was conducted during stable illumination periods near solar noon on June 6, June 20, June 30, July 15, July 22, August 15, August 23, and September 13, 2025. Before each flight, images of a standard reflectance calibration panel were collected for radiometric correction. In addition, ten 60 cm × 60 cm ground control targets were evenly placed within the experimental area to assist image identification, georeferencing and co-registration.

The original-resolution images were imported into Pix4Dmapper for preprocessing. For the multispectral imagery, radiometric calibration was first performed, followed by orthomosaic generation and geometric registration. For the thermal infrared imagery, radiometric processing was used to derive canopy temperature, and the thermal orthomosaic was spatially registered to the multispectral orthomosaic. The spatial resolution of the original orthomosaic images was approximately 6.48 cm. Based on the plot boundaries, plot regions were clipped, and canopy pixels were extracted after vegetation masking.

Vegetation masks were generated from the multispectral orthomosaics using NDVI threshold segmentation to remove background pixels such as soil and plastic mulch. Instead of applying a fixed threshold, a date-specific dynamic NDVI threshold was determined using Otsu’s method for each flight date, because canopy coverage varied across growth stages. Pixels with NDVI values greater than or equal to the threshold were retained as maize canopy pixels, whereas the remaining pixels were excluded as background. Morphological opening and closing operations were then applied to reduce isolated noisy pixels and small holes in the binary mask. The cleaned mask was applied to both the multispectral and thermal infrared orthomosaics for the extraction of spectral indices, canopy temperature, CWSI, and pixel-distribution-based features. Co-registration between the multispectral and thermal infrared orthomosaics was checked using ground control targets. After co-registration, the residual spatial offset between the two image datasets was generally within approximately one pixel of the common orthomosaic grid. The date-specific Otsu NDVI thresholds and canopy-mask keep ratios are provided in **Supplementary Table 2**.

### 2.3 Research methods

#### 2.3.1 Feature extraction

For each plot, pixel-level spectral, thermal, and water-stress-related features were constructed from the multispectral and thermal infrared imagery. After vegetation masking, only effective maize canopy pixels within each plot were used for feature extraction. Within-plot heterogeneity in UAV-derived canopy signals was characterized using the value distributions of these effective canopy pixels within each plot. Based on these pixel-level variables, plot-level statistical features, including mean, percentile, and dispersion features, were further extracted for subsequent correlation analysis, feature selection, and CWC estimation modeling. Specifically, the multispectral imagery was used to calculate typical vegetation indices, including the normalized difference vegetation index (NDVI), green normalized difference vegetation index (GNDVI), normalized difference red edge index (NDRE), and canopy chlorophyll content index (CCCI). These indices were used to characterize canopy vigor, chlorophyll status, and differences in vegetation growth. The thermal infrared imagery was used to extract canopy temperature (CT), from which the crop water stress index (CWSI) was further derived. The calculation formulas of the selected vegetation indices are shown in Equations 3–6.

(3) $$\mathrm{NDVI}=\frac{R_{\mathrm{NIR}}-R_{\mathrm{Red}}}{R_{\mathrm{NIR}}+R_{\mathrm{Red}}}$$

(4) $$\mathrm{GNDVI}=\frac{R_{\mathrm{NIR}}-R_{\mathrm{Green}}}{R_{\mathrm{NIR}}+R_{\mathrm{Green}}}$$

(5) $$\mathrm{NDRE}=\frac{R_{\mathrm{NIR}}-R_{\mathrm{RedEdge}}}{R_{\mathrm{NIR}}+R_{\mathrm{RedEdge}}}$$

(6) $$\mathrm{CCCI}=\frac{\mathrm{NDRE}}{\mathrm{NDVI}}$$

where R_NIR_ is the reflectance of the near-infrared band; R_Red_ is the reflectance of the red band; R_Green_ is the reflectance of the green band; and R_RedEdge_ is the reflectance of the red-edge band.

The thermal infrared imagery was used to extract canopy temperature (CT), from which the crop water stress index (CWSI) was further derived. After removing invalid thermal pixels, as calculated in Equation 7, CWSI was calculated for valid canopy pixels using an image-based empirical wet–dry reference method. For each flight date, Twet and Tdry were defined as the lower 0.2 percentile and upper 98.0 percentile of the masked canopy temperature distribution, respectively:

(7) $$\mathrm{CWSI}=\frac{T_{c}-T_{wet}}{T_{dry}-T_{wet}}$$

where ***T_c_*** is the canopy temperature of each valid canopy pixel. This CWSI was an image-based empirical index derived from the canopy-temperature distribution within each flight date, rather than a conventional meteorological-baseline CWSI based on non-stressed and fully stressed reference baselines defined using meteorological variables.

At the plot scale, effective canopy pixels within each plot were used as the statistical units to fully exploit the information contained in within-canopy pixel-value distributions. Plot-level statistical features were further extracted from the pixel-level variables. For NDVI, GNDVI, NDRE, and CCCI, the mean, 10th percentile (p10), standard deviation (sd), and interquartile range (iqr) were calculated. For CT and CWSI, the mean, standard deviation, and 90th percentile (p90) were calculated. The mean was used to represent the overall canopy condition. The lower and upper percentiles were used to describe abnormal information at the low-value and high-value ends of the pixel distribution, respectively. Standard deviation and interquartile range were used to characterize the dispersion of pixel values and within-plot canopy heterogeneity. Based on this procedure, a candidate feature pool consisting of mean features and distributional statistical features was constructed for subsequent correlation analysis, key feature identification, and CWC estimation modeling.

#### 2.3.2 Feature correlation analysis and selection

Considering the stage-dependent changes in maize canopy structure, leaf function, transpiration, and water-stress responses, the Shiyanghe samples were divided into pre-tasseling and post-tasseling stages based on field phenological observations. Tasseling was used as the division point because it marks the transition from vegetative to reproductive growth. According to the field records, 2025-07–11 was used as the analytical cutoff date: observations on 2025-06-06, 2025-06-20, and 2025-06–30 were assigned to the pre-tasseling stage, whereas observations on 2025-07-15, 2025-07-22, 2025-08-15, 2025-08-23, and 2025-09–13 were assigned to the post-tasseling stage.

Within each stage, feature selection was embedded in the repeated train–test splitting framework. In each repetition, RFECV was performed only within the training set to prevent information from the testing set from entering the feature selection process. The training set was used for feature selection and model training, whereas the testing set was used only for final evaluation. Recursive feature elimination with cross-validation (RFECV) was used to identify the key features for each growth stage. A random forest regressor was used as the base estimator in RFECV. Under five-fold cross-validation, features were recursively eliminated, and the optimal feature subset was determined based on the minimum cross-validated root mean square error (RMSE). The selected feature subsets were then used for subsequent stage-specific CWC estimation modeling.

#### 2.3.3 Model development and evaluation

To evaluate the ability of different feature sets to characterize maize CWC, CWC estimation models were developed separately for the pre-tasseling and post-tasseling stages. Ground-measured CWC was used as the target variable, and two types of input feature sets were defined. The first was the mean-only feature set, including NDVI_mean, GNDVI_mean, NDRE_mean, CCCI_mean, CT_mean, and CWSI_mean. The second was the RFECV-selected feature set. By comparing model prediction performance under different feature sets, the contributions of mean features and distributional statistical features to CWC estimation were assessed.

Four regression methods were used for model development: gradient boosting decision tree (GBDT), partial least squares regression (PLSR), random forest regression (RFR), and support vector regression (SVR). To evaluate model robustness and ensure comparability among feature sets, model development was conducted using 50 repeated random train–test splits within each growth stage. In each repetition, 80% of the samples were used for training and the remaining 20% were used as a held-out testing set. Feature selection and hyperparameter optimization were performed only within the training set, and the testing set was used only for final evaluation. Hyperparameters were optimized using five-fold cross-validation within the training set and a two-stage random search strategy, with the optimal parameters determined by the minimum cross-validated RMSE. The main hyperparameters, candidate search ranges, and optimization settings for the four regression models are summarized in **Supplementary Table 3**. Model performance was evaluated using the coefficient of determination (R²) and root mean square error (RMSE), as calculated in Equations 8 and 9, and the results from the 50 repetitions were summarized as mean ± standard deviation. To compare the mean-only and RFECV-selected feature sets, paired Wilcoxon signed-rank tests were performed on the paired R² and RMSE values obtained from the same 50 train–test splits.

(8) $$R^{2}=1-\frac{\sum_{i=1}^{n}{\left({\hat{y}}_{i}-y_{i}\right)}^{2}}{\sum_{i=1}^{n}{\left({\bar{y}}_{i}-y_{i}\right)}^{2}}$$

(9) $$RMSE=\sqrt{\frac{\sum_{i=1}^{n}{\left(y_{i}-{\hat{y}}_{i}\right)}^{2}}{n}}$$

where y_i_ is the observed maize CWC, ${\hat{y}}_{i}$ is the model−predicted value, $\bar{y}\;$ is the mean observed CWC, and ***n*** is the total number of samples.

#### 2.3.4 Spatial aggregation and scale-effect analysis

Because reduced spatial resolution can smooth local differences within the canopy and thereby affect the identification of temperature variation, spectral changes, and water stress, it is necessary to compare information retention and its influence on model performance across different scales within a consistent image-processing framework. To analyze the effects of spatial resolution changes on the characterization of maize canopy heterogeneity under water stress, the original-resolution multispectral reflectance bands and canopy-temperature orthomosaic images derived from thermal infrared imagery were used as the basis for spatial aggregation. Mean resampling was applied with aggregation windows of 2 × 2, 4 × 4 and 8 × 8 to generate reconstructed images at different spatial resolutions. Consequently, the actual spatial resolutions of the aggregated multispectral images and thermal images progressed from the original 6.48 cm/pixel to 12.96 cm/pixel, 25.92 cm/pixel, and 51.84 cm/pixel, respectively. If the original spatial resolution is *R*_0_, the reconstructed resolution is $R_{n}=nR_{0}(n=2,\;4,\;8)$. At aggregation scale s, the value of each reconstructed pixel was determined by the mean value of the corresponding pixels within the original neighborhood, as shown in Equation 10.

(10) $$I_{u,\;v,\;c}^{\left(s\right)}=\frac{1}{\left|\Omega_{u,\;v}^{\left(s\right)}\right|}\underset{\left(i,\;j\right)\in \Omega_{u,\;v}^{\left(s\right)}}{\sum }I_{i,\;j,\;c}$$

Where *I_i_*, *_j_*, *_c_* is the pixel value of band c at position (*i*, *j*) in the original image, and ${\text{Ω}}_{u,\;v}^{\left(s\right)}$ denotes the original-scale neighborhood corresponding to the reconstructed pixel (*u*, *v*).

Crucially, spatial aggregation was strictly applied to the original spectral reflectance bands and canopy temperature data. During reconstruction, image dimensions and spatial transformation parameters were updated simultaneously, while the coordinate reference system was kept unchanged. This ensured spatial correspondence among images at different scales and between the images and plot boundaries. Under the same spatial extent and coordinate reference system, resolution degradation was simulated by progressively increasing pixel size. At each reconstructed scale, the same processing workflow as that used for the original images was applied. Effective canopy pixels were extracted using the same plot boundaries, and plot-level features were recalculated, including multispectral vegetation indices, canopy temperature, CWSI, and their statistical features. Based on these reconstructed datasets, the relationships between features and CWC, as well as changes in model performance, were compared across spatial aggregation scales. This analysis was used to evaluate the effects of simulated spatial aggregation on feature stability and CWC estimation accuracy and to identify key features that were either sensitive or robust to changes in spatial scale.

## 3 Results

### 3.1 Remote sensing responses of maize canopy under water stress

#### 3.1.1 Stage-dependent differentiation of CWC under different irrigation treatments

**Figure 4:**
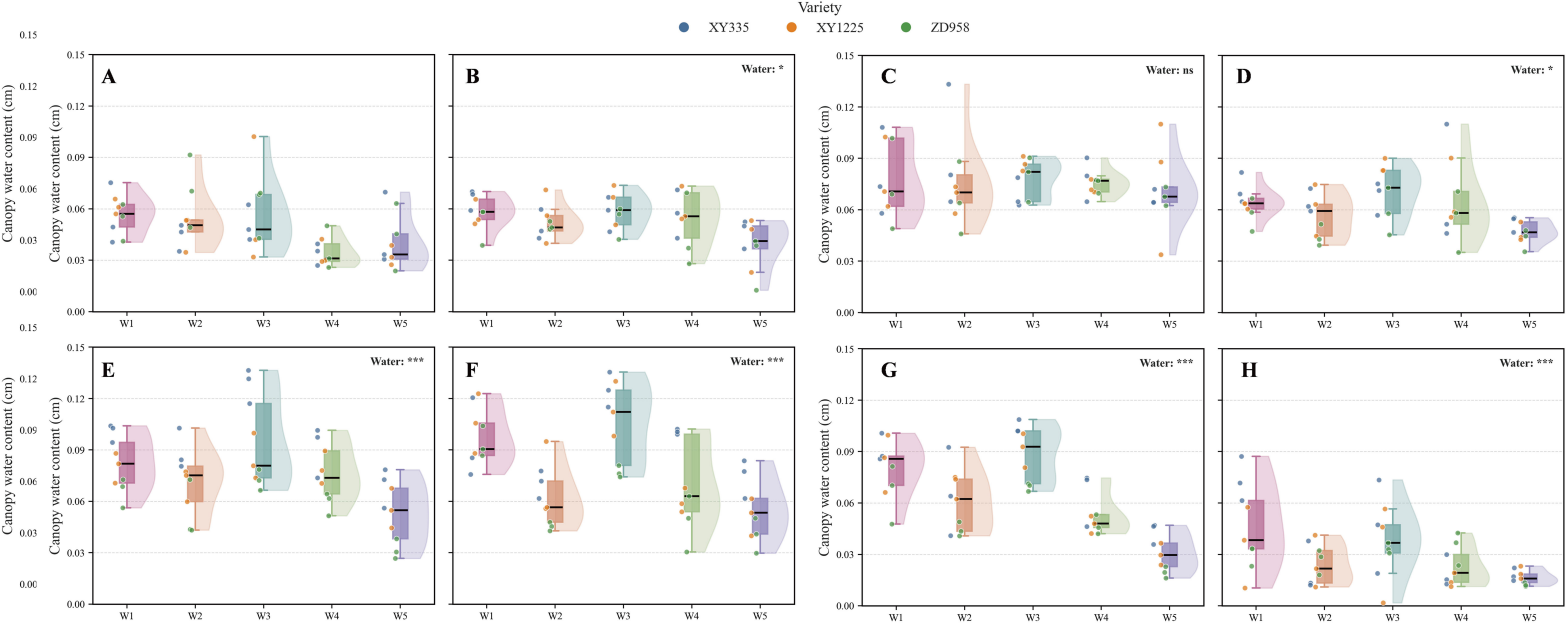
Changes in maize canopy water content under different growth stages and irrigation treatments at the Shiyanghe site. Panels **(A–H)** represent the seedling stage, jointing stage, trumpet stage, tasseling stage, silking stage, middle grain-filling stage, late grain-filling stage, and maturity stage, respectively. Asterisks indicate the overall significance of the main effect of irrigation treatment within each observation date, as determined by two-way analysis of variance. ns, *, **, and *** indicate p ≥ 0.05, p< 0.05, p< 0.01, and p< 0.001, respectively. The seedling-stage data at the Shiyanghe site were collected before irrigation treatments were imposed and are shown only as a baseline; therefore, no significance annotation is provided.

The CWC data collected at the seedling stage at the Shiyanghe site were obtained before the irrigation treatments were imposed and were therefore not interpreted as treatment effects. As shown in **Figure 4**, after maize entered the jointing stage, CWC began to show some differentiation among irrigation treatments, and the main effect of irrigation became evident (p = 0.0174). Specifically, higher-water-supply treatments, such as W1 and W3, generally maintained higher CWC, whereas lower-water-supply treatments, such as W4 and W5, showed relatively lower values. However, the distributions among treatments still overlapped considerably at this stage, indicating limited treatment separation.

At the late vegetative stage before tasseling, here referred to as the trumpet stage, the main effect of irrigation was not significant (p = 0.9356), suggesting that CWC differences among treatments temporarily weakened. This indicates that the response of canopy water content to water deficit did not increase monotonically with crop development but showed stage-dependent fluctuations. From the tasseling stage onward, significant differences among irrigation treatments reappeared (p = 0.0129) and became more pronounced after the silking stage (p< 0.001). In particular, during the silking stage, middle grain-filling stage, and late grain-filling stage, full or higher-water-supply treatments, such as W1 and W3, generally maintained higher canopy water content, whereas deficit treatments, especially W5, showed a clear downward shift. The overlap among treatment distributions decreased markedly, indicating that a stable and distinct CWC gradient was formed at the Shiyanghe site during the middle and late growth stages. Notably, compared with whole-season deficit irrigation, regulated deficit irrigation maintained canopy water content more effectively while saving water. By the maturity stage, however, CWC decreased rapidly across all treatments and tended to converge.

The distribution of cultivar-level scatter points showed that, although some dispersion among cultivars within the same treatment remained during the middle and late growth stages at the Shiyanghe site, most observations were clustered around their respective treatment levels. This suggests that the direction of the main irrigation effect was generally consistent across cultivars.

#### 3.1.2 Spatial distribution characteristics of CWSI under different irrigation treatments

**Figure 5:**
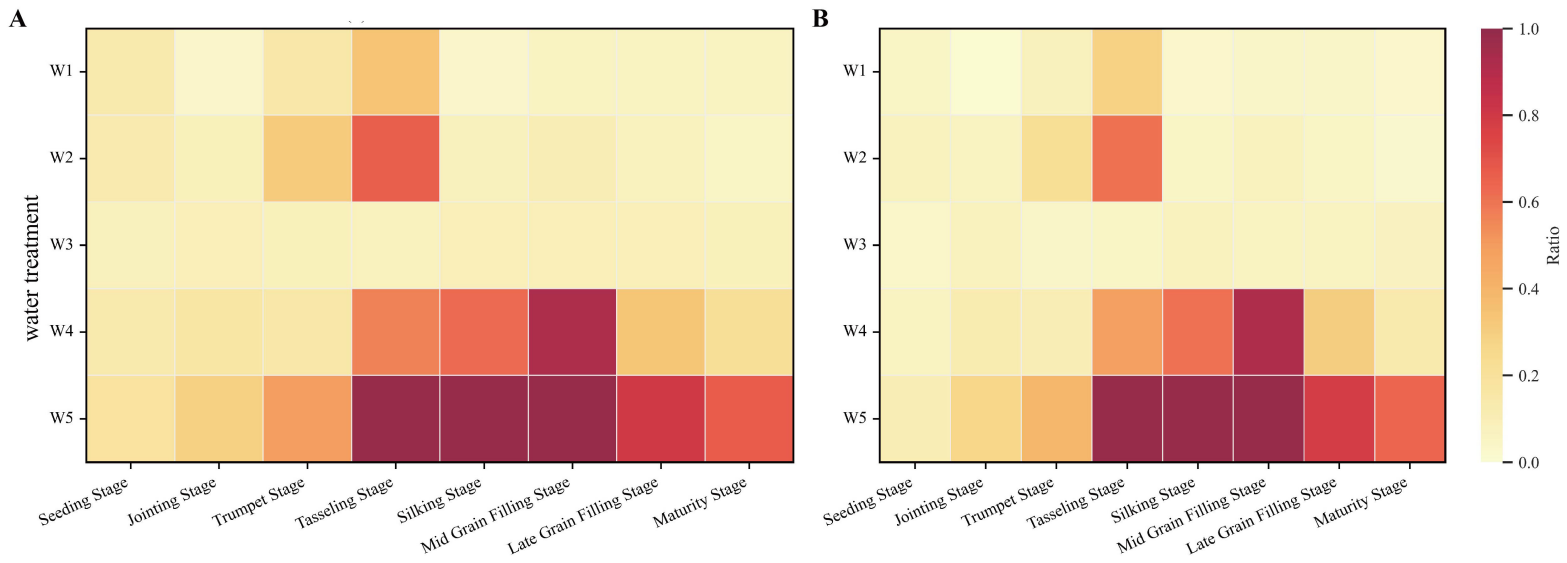
Heatmaps of the anomaly area ratio and largest patch ratio of CWSI under different irrigation treatments. **(A)** Anomaly area ratio; **(B)** largest patch ratio.

As shown in **Figure 5**, a stable and clear differentiation in CWC among irrigation treatments had already formed at the Shiyanghe site from the silking stage to the late grain-filling stage. To quantify the expansion of thermally anomalous areas within plots, this study used the 90th percentile of the CWSI pixel distribution in the W3 treatment on the same observation date as the threshold for identifying relatively high-stress pixels. Based on this threshold, two metrics were calculated: the anomaly area ratio and the largest patch ratio. The anomaly area ratio represents the proportion of anomalous pixels relative to the effective canopy area, whereas the largest patch ratio represents the proportion of the largest connected anomalous patch relative to the effective canopy area.

The heatmaps of the CWSI anomaly area ratio and largest patch ratio showed clear stage-dependent and treatment-gradient patterns in the spatial expansion of thermally anomalous pixels. Under the full irrigation treatment W3, both metrics remained generally low throughout the growing season, indicating that high-CWSI anomalous pixels were mainly distributed in small, scattered areas and that the canopy spatial pattern remained relatively stable.

In contrast, under the whole-season deficit treatments W4 and W5, both the anomaly area ratio and the largest patch ratio increased markedly from the tasseling stage onward and remained high from the post-tasseling stage through the grain-filling period. Among these treatments, W5 showed the highest values of both metrics at the tasseling stage, silking stage, and the middle and late grain-filling stages, indicating that anomalous regions not only expanded in coverage but also developed from localized point-like distributions into larger patches. The trend for W4 was generally similar to that of W5, although the overall intensity was slightly lower, suggesting that even mild but continuous deficit throughout the growing season was sufficient to induce the aggregation and expansion of thermally anomalous pixels.

By comparison, W1 and W2 showed only limited increases at a few growth stages, and both their persistence and spatial expansion were clearly weaker than those of W4 and W5. This suggests that regulated deficit irrigation was more effective in suppressing the large-scale expansion of anomalously high-temperature regions and in maintaining a relatively stable canopy structure.

**Figure 6:**
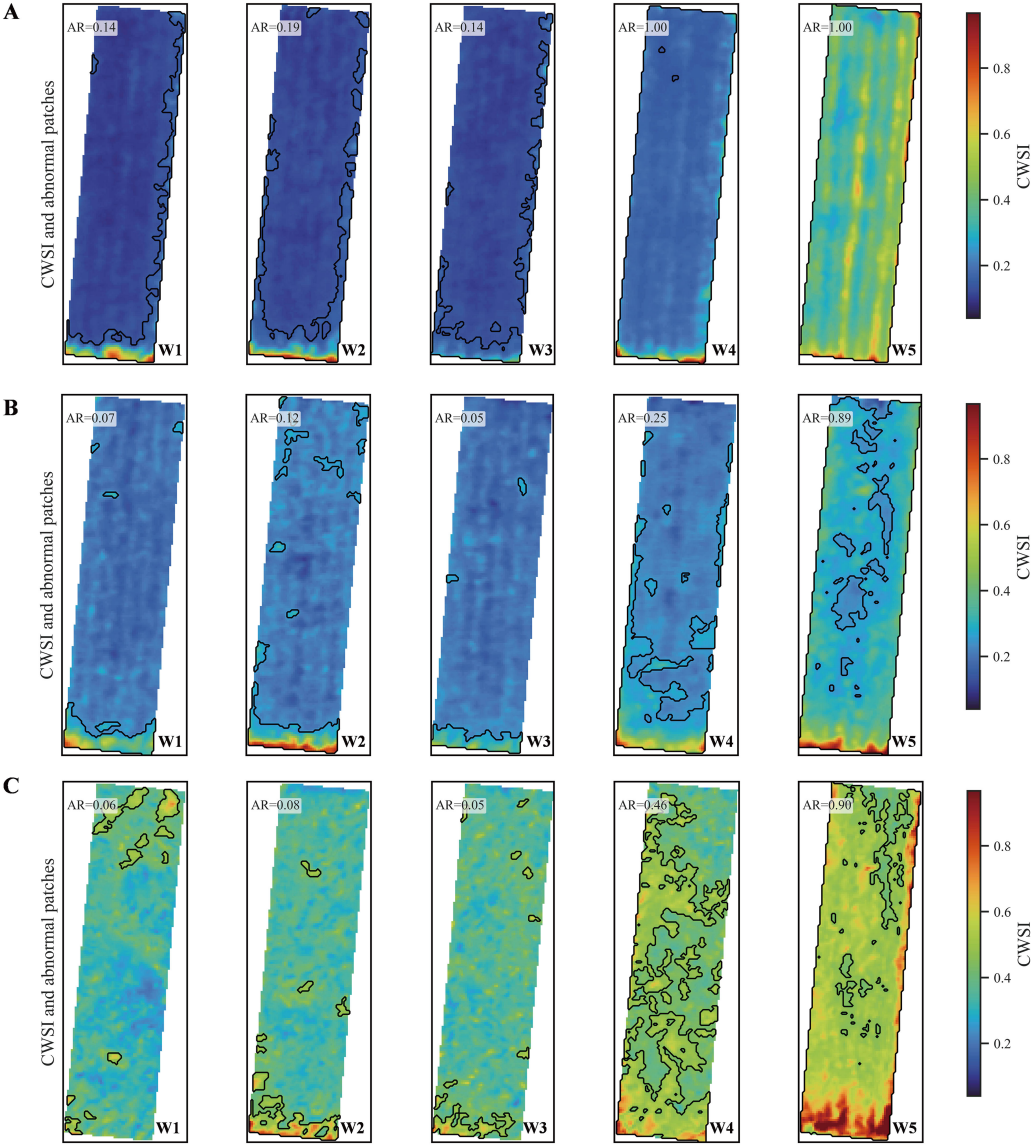
Spatial distribution of maize canopy CWSI and high-value anomalous patches in fixed plots of the same cultivar at the Shiyanghe site under different growth stages and irrigation treatments. Panels **(A–C)** show the CWSI distributions at the tasseling–silking stage (2025-07-22), grain-filling stage (2025-08-23), and maturity stage (2025-09-13), respectively. AR denotes the proportion of the high-CWSI anomalous area relative to the effective canopy area.

To eliminate the potential influence of changing representative plots across observation dates, a further cross-temporal comparison was conducted using the same cultivar (ZD958) and the same set of treatment plots to examine the spatial distribution of maize canopy CWSI and the evolution of anomalous patches. As shown in **Figure 6**, the spatial pattern of thermal anomalies under different irrigation treatments began to diverge clearly after the tasseling stage and persisted through the late grain-filling and maturity stages. At the tasseling–silking stage, the W3 treatment was still dominated by low CWSI values, with few anomalous patches and a scattered distribution, indicating relatively uniform canopy thermal conditions under full irrigation. In contrast, more continuous anomalous regions had already appeared in W5, suggesting that under severe deficit treatment, localized high-temperature pixels had begun to expand from a scattered pattern into connected areas. These spatial patterns provide a basis for using pixel-distribution-based statistical features to summarize within-plot canopy-signal heterogeneity for subsequent plot-level CWC estimation. High-percentile thermal or CWSI features (e.g., CT_p90 and CWSI_p90) indicate the occurrence and expansion of localized high-temperature or high-stress canopy pixels, whereas dispersion features (sd and iqr) reflect within-plot variability and patch aggregation of these anomalous signals. For multispectral indices, lower-percentile and dispersion features capture increased variability and low-value pixels associated with uneven canopy vigor, localized senescence, or partial background exposure under water stress.

By the grain-filling stage (2025-08-23), the treatment differences became more pronounced. The anomalous regions in W4 and especially W5 expanded substantially, with AR increasing to 0.25 and 0.89, respectively, whereas W3 still maintained a low anomaly level (AR = 0.05). At the maturity stage (2025-09-13), anomalous regions in W4 and W5 still occupied large areas and showed stronger aggregation, with AR values of 0.45 and 0.90, respectively. In contrast, W1, W2, and W3 remained at relatively low levels, with AR values of 0.06, 0.08, and 0.05, respectively.

Taken together, the spatial maps and the patch-change heatmaps indicate that water stress was not expressed as a synchronous and uniform deterioration across the entire canopy. Instead, it first appeared as localized high-CWSI anomalous pixels and then, during the middle and late growth stages—especially under more severe deficit treatments—expanded into larger anomalous patches. This suggests that the enhancement of canopy thermal-signal heterogeneity was mainly expressed through two processes: the expansion of anomalous-area coverage and the increase in patch aggregation.

Consistent with the expansion of high-CWSI anomalous pixels in the thermal infrared domain, some multispectral vegetation indices, such as NDRE, also showed a certain increase in distributional heterogeneity during the middle and late growth stages, mainly manifested as a downward shift in the lower tail and an increase in within-plot dispersion. Overall, however, the treatment differentiation in multispectral distributional responses was weaker than that in CWSI and appeared later, suggesting that thermal responses provided a relatively more sensitive spatial representation of water-stress differences under the present experimental conditions.

### 3.2 Relationships between statistical features and CWC and estimation results at the original resolution

#### 3.2.1 Correlation analysis between features and CWC and RFECV-based feature selection

**Figure 7:**
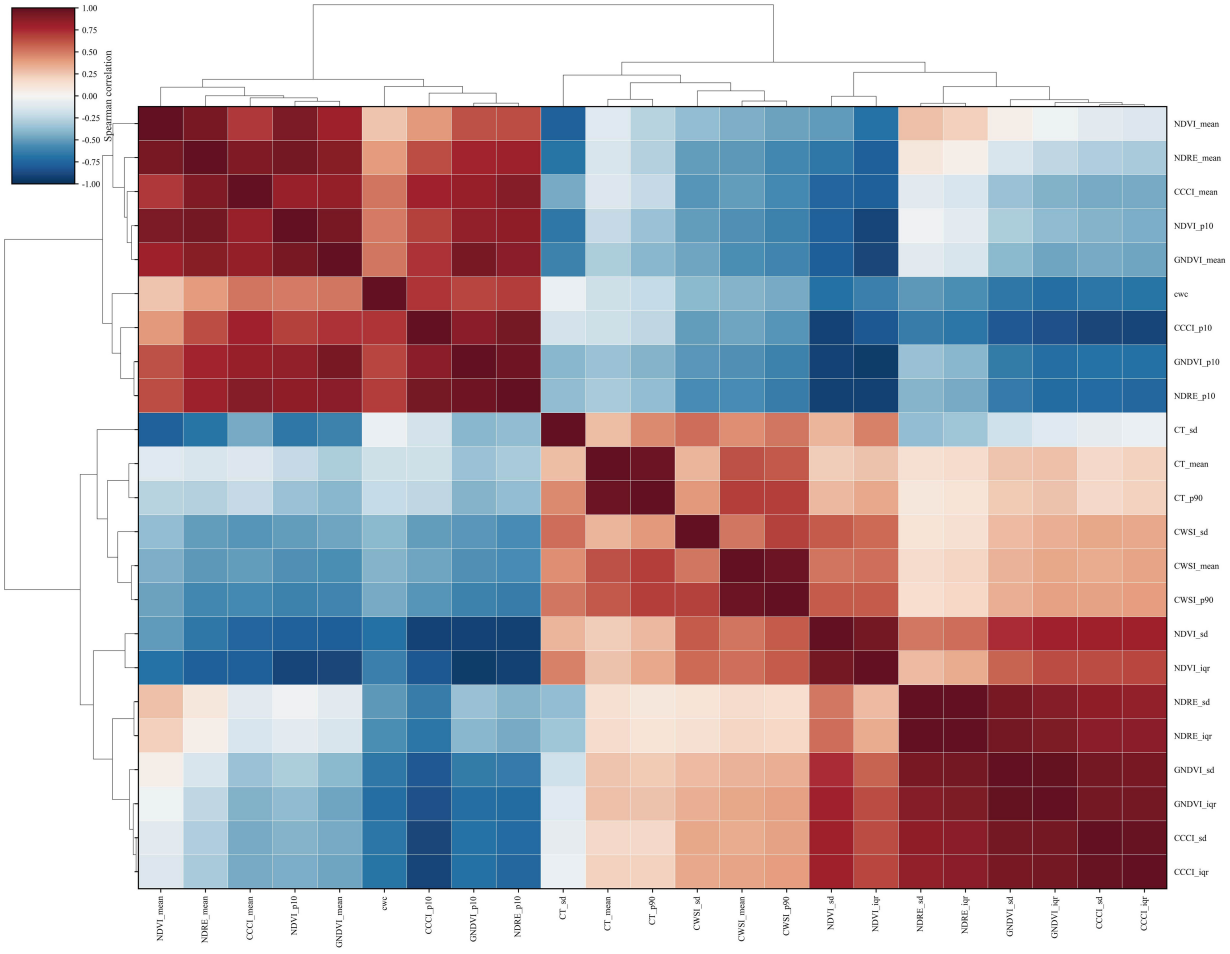
Spearman correlation heatmap between features and CWC.

Spearman’s rank correlation analysis was conducted between the candidate features extracted from the original-resolution imagery and observed CWC, and the results are shown in **Figure 7**. At the original resolution, the features showed a clear grouping structure. Multispectral mean features and lower-percentile features were generally clustered together and were positively correlated with CWC, indicating that both the mean level of canopy spectral responses and lower-tail information could reflect maize canopy water status.

Thermal and water-stress-related features formed another group and were generally negatively correlated with CWC and multispectral mean features. This suggests that higher canopy temperature and stronger water-stress signals were generally associated with lower canopy water content. Meanwhile, dispersion features of vegetation indices, including sd and iqr, formed a relatively independent group. These features showed high consistency with thermal and stress-related features but were generally negatively correlated with CWC and multispectral mean features. This suggests that within-plot canopy heterogeneity was closely associated with crop thermal status and water stress intensity. Overall, distributional statistical features at the original resolution showed an information structure distinct from that of mean features.

To reveal growth-stage differences in the relationships between canopy features and CWC, CWC estimation models were developed separately for the pre-tasseling and post-tasseling stages. Because RFECV-selected features may vary with random data partitioning, RFECV feature selection was repeated within the training set during the 50 repeated random splits. The selection frequency of each feature was then used to evaluate the stability of RFECV-selected features.

**Figure 8:**
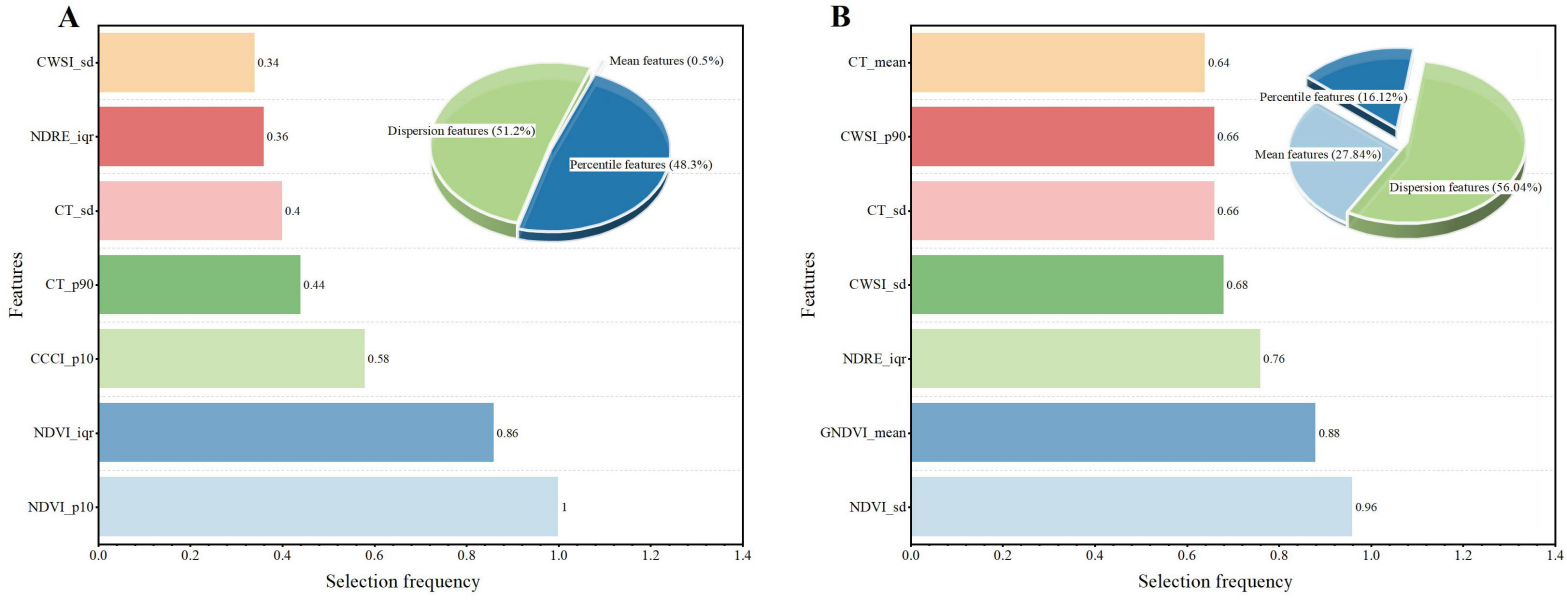
RFECV feature-selection frequencies from 50 repeated random train–test splits. **(A)** Pre-tasseling stage; **(B)** post-tasseling stage. Bars show the selection frequency of individual features, and inset donut charts show the proportions of mean, percentile, and dispersion features calculated from the total RFECV selection counts. The proportions do not represent feature-importance values.

The RFECV feature-selection frequencies obtained from the 50 repeated random splits are shown in **Figure 8**. The RFECV feature-selection frequency showed clear growth-stage dependence. Before tasseling, the selected features were dominated by percentile and dispersion features, with NDVI_p10, NDVI_iqr, and CCCI_p10 showing the highest selection frequencies. Dispersion and percentile features accounted for 51.18% and 48.34% of the total selection counts, respectively, whereas mean features accounted for only 0.47%. These proportions were calculated from the total RFECV selection counts rather than from feature-importance values. This indicates that, before tasseling, RFECV feature selection was more frequently associated with lower-tail canopy responses and within-plot heterogeneity.

After tasseling, both distributional and mean features were selected. High-frequency features included NDVI_sd, GNDVI_mean, NDRE_iqr, CWSI_sd, CT_sd, CWSI_p90, and CT_mean. The proportion of mean features increased to 27.84%, suggesting that overall canopy status became more involved after tasseling; however, dispersion features still accounted for the largest proportion of the total selection counts.

#### 3.2.2 CWC estimation results at the original resolution

For the stage-specific CWC estimation results at the original resolution, the best-performing models and the effects of feature sets differed between the pre-tasseling and post-tasseling stages, as shown in **Table 1**. Model performance was evaluated based on 50 repeated random train–test splits. The repeated random-split results indicate that heterogeneity-related features contributed to CWC estimation in a stage- and model-dependent manner at the original resolution, and the scale dependence of feature–CWC associations and model performance is further examined in Section 3.3.

**Table 1:** Robustness and statistical comparison of model performance based on repeated random splits.

Growth stage	Model	Feature set	R² mean ± SD	RMSE mean ± SD (cm)	Wilcoxon p value for ΔR²	Wilcoxon p value for ΔRMSE	Better significance set
Pre-tasseling	GBDT	Mean-only	0.332 ± 0.217	0.01593 ± 0.00318	<0.001	<0.001	×
		RFECV-selected	0.532 ± 0.146	0.01482 ± 0.00269			✓
	RFR	Mean-only	0.371 ± 0.192	0.01551 ± 0.00295	<0.001	<0.001	×
		RFECV-selected	0.529 ± 0.160	0.01482 ± 0.00280			✓
	PLSR	Mean-only	0.393 ± 0.170	0.01528 ± 0.00276	0.901	0.752	ns
		RFECV-selected	0.387 ± 0.160	0.01536 ± 0.00251			ns
	SVR	Mean-only	0.430 ± 0.186	0.01474 ± 0.00313	0.009	0.010	✓
		RFECV-selected	0.381 ± 0.172	0.01542 ± 0.00266			×
Post-tasseling	GBDT	Mean-only	0.445 ± 0.087	0.02037 ± 0.00241	0.901	0.818	ns
		RFECV-selected	0.444 ± 0.093	0.02035 ± 0.00225			ns
	RFR	Mean-only	0.468 ± 0.112	0.01988 ± 0.00242	0.040	0.042	✓
		RFECV-selected	0.446 ± 0.113	0.02028 ± 0.00242			×
	PLSR	Mean-only	0.453 ± 0.097	0.02018 ± 0.00224	<0.001	<0.001	✓
		RFECV-selected	0.425 ± 0.099	0.02068 ± 0.00215			×
	SVR	Mean-only	0.453 ± 0.086	0.02023 ± 0.00241	0.709	0.716	ns
		RFECV-selected	0.451 ± 0.090	0.02024 ± 0.00232			ns

During the pre-tasseling stage, the repeated random-split validation showed that the advantage of the RFECV-selected feature set was model-dependent rather than universal. The RFECV-selected feature set significantly improved the performance of GBDT and RFR, whereas no significant improvement was observed for PLSR, and the mean-only feature set performed better for SVR. These results suggest that distribution-related features provided complementary information before tasseling, but their usefulness at the original resolution was more evident for tree-based models.

During the post-tasseling stage, the mean-only feature set showed equal or more stable performance across models. In particular, the mean-only feature set significantly outperformed the RFECV-selected feature set for PLSR and RFR, while the differences for GBDT and SVR were not statistically significant. This indicates that, at the original resolution, post-tasseling CWC estimation was more closely associated with overall canopy-level spectral and thermal conditions, whereas the additional contribution of distribution-related features was limited or model-dependent.

**Figure 9:**
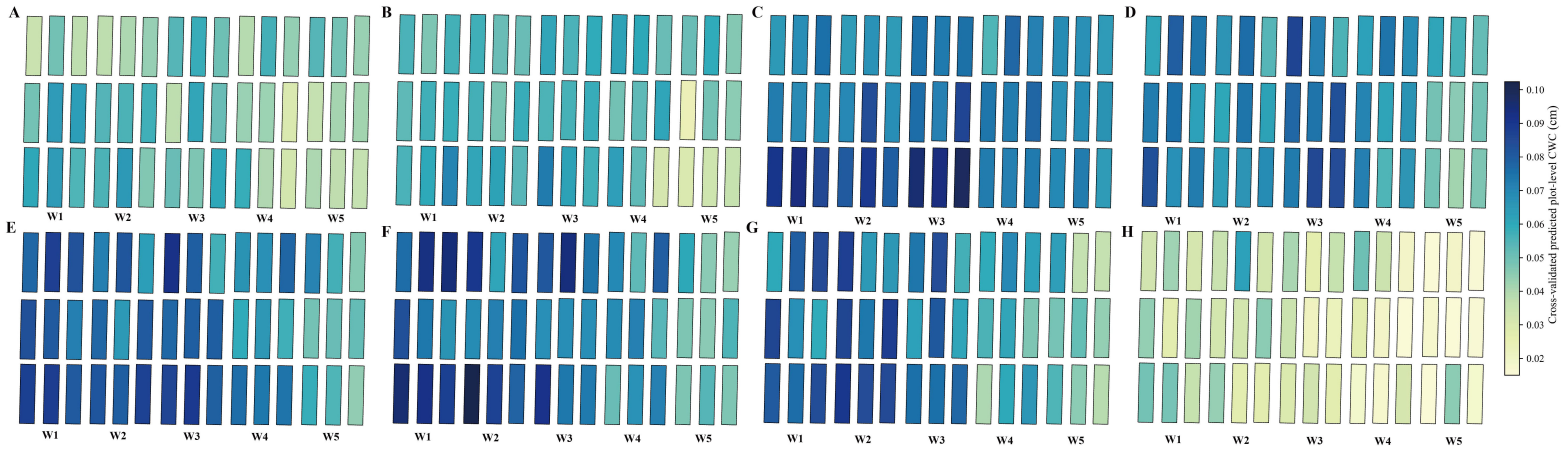
Cross-validated plot-level spatial distribution of predicted CWC at the Shiyanghe site across eight flight dates. For each plot-date observation, the mapped value represents the mean test-set prediction obtained across the 50 repeated random splits. **(A)** 2025-06-06; **(B)** 2025-06-20; **(C)** 2025-06-30; **(D)** 2025-07-15; **(E)** 2025-07-22; **(F)** 2025-08-15; **(G)** 2025-08-23; **(H)** 2025-09-13. W1–W5 indicate irrigation treatments. These maps represent plot-level CWC predictions rather than pixel-scale CWC retrievals.

**Figure 9** shows the cross-validated plot-level spatial distribution of predicted CWC at the Shiyanghe site. For each plot-date observation, the mapped value represents the mean test-set prediction obtained across the 50 repeated random splits. Overall, the predicted CWC showed a clear temporal pattern. The values were relatively low during the early growth stage, increased from late June to the tasseling–silking and grain-filling stages, and then decreased markedly at maturity. This temporal pattern was generally consistent with the seasonal dynamics of ground-measured CWC.

### 3.3 Effects of spatial aggregation scale on statistical features and model performance

#### 3.3.1 Scale responses of representative high-frequency RFECV-selected features under spatial aggregation

**Figure 10:**
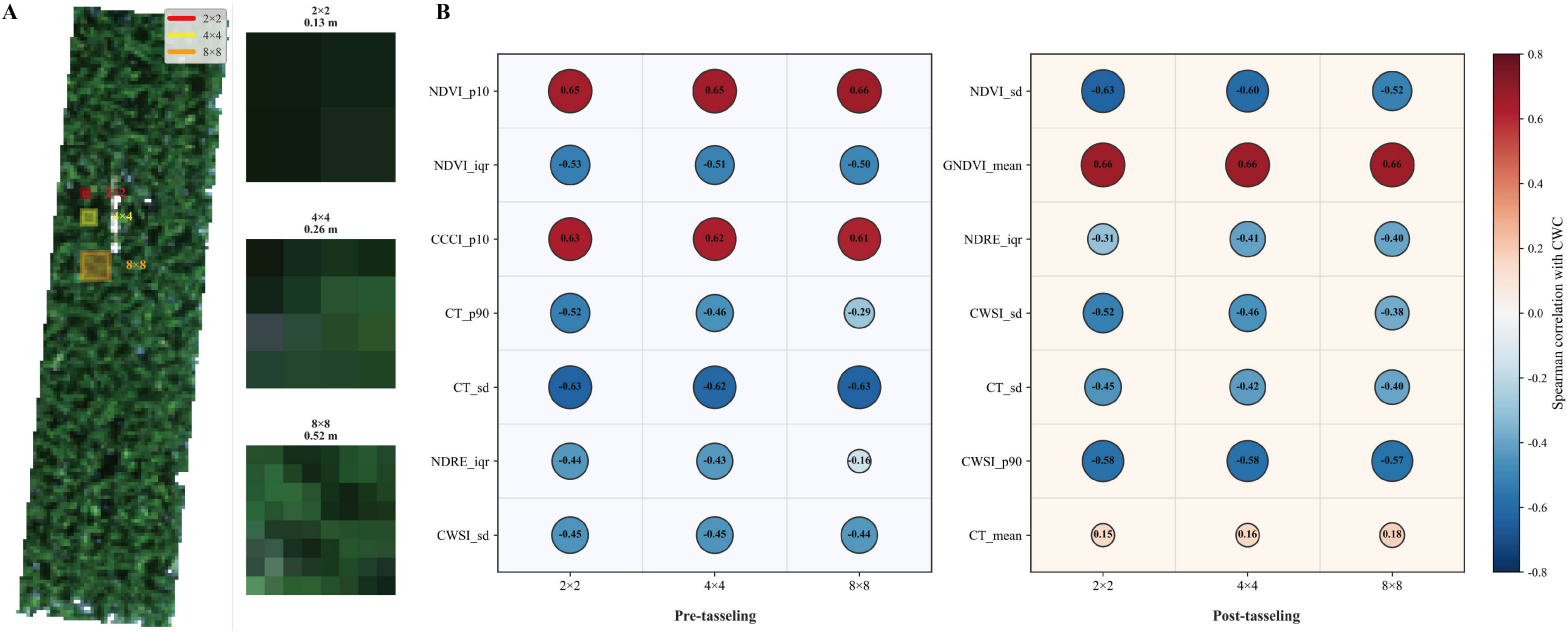
Scale responses of the relationships between RFECV-selected key features and CWC. **(A)** Schematic diagram of the spatial aggregation scales; **(B)** changes in the Spearman correlation coefficients between key features and CWC across different spatial aggregation scales.

To maintain consistency with the repeated feature-selection framework at the original resolution, the features analyzed in this section were representative high-frequency RFECV-selected features identified from the 50 repeated random splits, rather than features obtained from a single RFECV run. As shown in **Figure 10**, most of these representative features maintained the same correlation direction with CWC across the 2 × 2, 4 × 4, and 8 × 8 aggregation scales. This indicates that simulated spatial aggregation mainly affected the strength of feature–CWC associations, rather than reversing their correlation directions.

During the pre-tasseling stage, lower-percentile and dispersion-related features retained strong CWC-related correlations across scales. NDVI_p10 and CCCI_p10 showed stable positive correlations with CWC, whereas NDVI_iqr, CT_sd, and CWSI_sd maintained stable negative correlations. However, not all distributional features responded similarly to spatial aggregation. CT_p90 and NDRE_iqr were more sensitive to coarser aggregation, especially at the 8 × 8 scale, where their correlations with CWC were clearly weakened. During the post-tasseling stage, GNDVI_mean showed a stable positive correlation with CWC across all aggregation scales, suggesting that overall canopy greenness remained a robust indicator of CWC variation after tasseling. In addition, CWSI_p90 maintained a stable negative correlation, while NDVI_sd, CWSI_sd, and CT_sd remained negatively correlated with CWC but showed different degrees of attenuation with increasing aggregation scale. These results indicate that heterogeneity-related features still contained CWC-related information after tasseling, but their scale responses were feature-specific rather than uniformly weakened.

Overall, the cross-scale correlation analysis showed that representative high-frequency RFECV-selected features generally retained their correlation directions under simulated spatial aggregation, but the magnitude of their correlations varied among features. The predictive contribution of these features was further evaluated through repeated model comparisons in Section 3.3.2, where model type, growth stage, and aggregation scale jointly determined whether RFECV-selected features provided additional predictive value beyond mean-only features.

#### 3.3.2 Comparison of CWC estimation performance across spatial aggregation scales

In this subsection, the RFECV-selected feature set refers to the feature subset selected within the repeated modeling procedure at each aggregation scale, rather than the fixed representative features tracked in Section 3.3.1. The mean-only and RFECV-selected feature sets were compared at the 2 × 2, 4 × 4, and 8 × 8 aggregation scales using four regression models. Model performance was summarized from 50 repeated random splits, and paired Wilcoxon tests were used to compare the two feature sets under each growth stage, aggregation scale, and model, as shown in **Table 2**. During the pre-tasseling stage, the relative performance of the two feature sets changed clearly with aggregation scale. At the 2 × 2 scale, the RFECV-selected feature set achieved higher mean R² and lower RMSE than the mean-only feature set for all four models, and the differences were significant. This indicates that, under the repeated-split evaluation used in this study, within-plot pixel distribution information provided additional predictive value at the 2 × 2 scale. At the 4 × 4 scale, the feature-set effect became model-dependent: RFECV-selected features improved the performance of RFR and SVR, whereas PLSR performed better with mean-only features, and GBDT showed no significant difference. At the 8 × 8 scale, the pattern was reversed, with the mean-only feature set outperforming the RFECV-selected feature set across all four models.

**Table 2:** Effects of UAV pixel-level feature aggregation strategies on plot-scale CWC estimation across growth stages, spatial aggregation scales, and regression models.

Growth stage	Spatial scale	Model	Feature set	R² mean ± SD	RMSE mean ± SD (cm)	Wilcoxon *p* value for ΔR²	Wilcoxon *p* value for ΔRMSE	Better significance set
Pre-tasseling	2×2	GBDT	Mean-only	0.299 ± 0.196	0.01641 ± 0.00247	0.002	0.004	×
			RFECV-selected	0.354 ± 0.184	0.01578 ± 0.00284			✓
		RFR	Mean-only	0.297 ± 0.211	0.01639 ± 0.00248	<0.001	<0.001	×
			RFECV-selected	0.386 ± 0.188	0.01537 ± 0.00288			✓
		PLSR	Mean-only	0.288 ± 0.239	0.01650 ± 0.00278	<0.001	<0.001	×
			RFECV-selected	0.384 ± 0.210	0.01532 ± 0.00254			✓
		SVR	Mean-only	0.288 ± 0.226	0.01647 ± 0.00259	0.002	0.004	×
			RFECV-selected	0.341 ± 0.215	0.01591 ± 0.00306			✓
	4×4	GBDT	Mean-only	0.356 ± 0.171	0.01577 ± 0.00262	0.146	0.198	ns
			RFECV-selected	0.328 ± 0.221	0.01600 ± 0.00253			ns
		RFR	Mean-only	0.330 ± 0.217	0.01596 ± 0.00236	0.004	0.006	×
			RFECV-selected	0.380 ± 0.192	0.01542 ± 0.00282			✓
		PLSR	Mean-only	0.380 ± 0.210	0.01536 ± 0.00256	<0.001	<0.001	✓
			RFECV-selected	0.306 ± 0.235	0.01628 ± 0.00279			×
		SVR	Mean-only	0.228 ± 0.261	0.01712 ± 0.00280	<0.001	<0.001	×
			RFECV-selected	0.340 ± 0.210	0.01591 ± 0.00293			✓
	8×8	GBDT	Mean-only	0.381 ± 0.192	0.01543 ± 0.00291	0.003	0.004	✓
			RFECV-selected	0.334 ± 0.194	0.01599 ± 0.00259			×
		RFR	Mean-only	0.394 ± 0.180	0.01527 ± 0.00282	0.003	0.004	✓
			RFECV-selected	0.347 ± 0.196	0.01581 ± 0.00260			×
		PLSR	Mean-only	0.378 ± 0.200	0.01540 ± 0.00241	<0.001	<0.001	✓
			RFECV-selected	0.327 ± 0.222	0.01605 ± 0.00285			×
		SVR	Mean-only	0.336 ± 0.207	0.01596 ± 0.00301	0.026	0.032	✓
			RFECV-selected	0.304 ± 0.220	0.01629 ± 0.00276			×
Post-tasseling	2×2	GBDT	Mean-only	0.476 ± 0.098	0.02020 ± 0.00215	0.004	0.003	✓
			RFECV-selected	0.447 ± 0.088	0.02078 ± 0.00195			×
		RFR	Mean-only	0.475 ± 0.104	0.02017 ± 0.00206	0.027	0.020	✓
			RFECV-selected	0.454 ± 0.095	0.02062 ± 0.00204			×
		PLSR	Mean-only	0.422 ± 0.090	0.02124 ± 0.00199	0.009	0.009	×
			RFECV-selected	0.437 ± 0.096	0.02095 ± 0.00217			✓
		SVR	Mean-only	0.404 ± 0.101	0.02156 ± 0.00221	<0.001	<0.001	×
			RFECV-selected	0.470 ± 0.090	0.02034 ± 0.00219			✓
	4×4	GBDT	Mean-only	0.466 ± 0.102	0.02037 ± 0.00211	<0.001	<0.001	✓
			RFECV-selected	0.429 ± 0.095	0.02110 ± 0.00195			×
		RFR	Mean-only	0.473 ± 0.105	0.02022 ± 0.00220	<0.001	<0.001	✓
			RFECV-selected	0.441 ± 0.101	0.02084 ± 0.00200			×
		PLSR	Mean-only	0.425 ± 0.088	0.02119 ± 0.00196	0.653	0.674	ns
			RFECV-selected	0.425 ± 0.099	0.02118 ± 0.00223			ns
		SVR	Mean-only	0.405 ± 0.099	0.02155 ± 0.00212	<0.001	<0.001	×
			RFECV-selected	0.464 ± 0.094	0.02045 ± 0.00228			✓
	8×8	GBDT	Mean-only	0.465 ± 0.100	0.02039 ± 0.00208	<0.001	<0.001	✓
			RFECV-selected	0.422 ± 0.104	0.02120 ± 0.00210			×
		RFR	Mean-only	0.475 ± 0.105	0.02018 ± 0.00213	<0.001	<0.001	✓
			RFECV-selected	0.430 ± 0.105	0.02103 ± 0.00206			×
		PLSR	Mean-only	0.422 ± 0.090	0.02123 ± 0.00197	0.265	0.226	ns
			RFECV-selected	0.417 ± 0.093	0.02133 ± 0.00208			ns
		SVR	Mean-only	0.401 ± 0.103	0.02162 ± 0.00234	<0.001	<0.001	×
			RFECV-selected	0.439 ± 0.095	0.02092 ± 0.00206			✓

The post-tasseling stage showed a different response pattern. For GBDT and RFR, the mean-only feature set generally produced higher R² and lower RMSE than the RFECV-selected feature set across the three aggregation scales, especially at the 4 × 4 and 8 × 8 scales. PLSR showed limited sensitivity to feature-set choice, with RFECV-selected features performing better only at the 2 × 2 scale and no significant differences at coarser scales. In contrast, SVR consistently benefited from the RFECV-selected feature set across all three aggregation scales. These results show that the contribution of RFECV-selected features after tasseling was strongly model-dependent. Overall, the benefit of RFECV-selected features was scale-, stage-, and model-dependent. They showed a consistent advantage at the 2 × 2 scale before tasseling, whereas mean-only features were more stable at the 8 × 8 scale and in several post-tasseling tree-based models.

## 4 Discussion

### 4.1 Growth stage dependent contribution of statistical features to CWC estimation under water stress

In this study, the contribution of statistical features to maize canopy water content (CWC) estimation exhibited clear growth-stage and model dependence. At the original spatial resolution, RFECV selection frequencies derived from 50 repeated random splits indicated that feature selection before tasseling was dominated by percentile and dispersion features, whereas mean features were selected much less frequently. This finding suggests that, before tasseling, CWC-related information was more strongly associated with lower-tail canopy responses and within-plot canopy-signal variability. However, the predictive advantage of these features was not consistent across models, indicating that their value should be regarded as complementary and model-dependent rather than inherently superior to mean features. This observation is consistent with the findings of Li et al. (2026), while the present study further evaluated the role of distributional-feature enhancement in UAV-based maize CWC estimation.

Water stress can reduce transpirational cooling and increase leaf temperature. However, this response is rarely expressed uniformly across the canopy. Instead, it is influenced by leaf position, radiation load, root-zone water availability, and local microclimatic conditions. Consequently, localized hotspots and areas with reduced spectral responses often emerge before canopy-wide changes become evident under water stress (González-Dugo et al., 2006). These localized responses may help explain why percentile and dispersion features were effective in characterizing early within-plot canopy-signal heterogeneity.

Results showed that the distributional features were associated with CWC and irrigation-induced spatial patterns. The selected features also provide insight why statistical features contributed to CWC estimation in this study. NDVI essentially reflects the contrast between high near-infrared reflectance and red-light absorption, and is more closely associated with overall canopy greenness and cover status. Therefore, under early stress conditions, its mean value may be insufficient to reveal localized degradation, whereas lower-percentile and dispersion features are more likely to capture the early emergence of low-vigor areas (Brewer et al., 2022). GNDVI and NDRE are related to chlorophyll status and red-edge responses, respectively, and are sensitive to physiological changes and subtle signal differences under high canopy cover. Therefore, before water stress appears as a clear shift in the overall canopy mean, statistical features such as interquartile range, standard deviation, and lower percentiles may capture the expansion of low-value areas and the increasing dispersion of pixel-value distributions within plots.

Previous UAV-based multispectral–thermal infrared studies on maize have shown that NDRE, GNDVI, CCCI, and thermal infrared variables are important factors for CWSI prediction, and that multivariable modeling helps extract effective information related to crop water status (Wei et al., 2021; Kapari et al., 2024). As an index combining red-edge information and vegetation cover information, CCCI can enhance the representation of chlorophyll variation to some extent. CT and CWSI represent the most direct thermal response and relative stress level, respectively, whereas their standard deviation and upper-percentile features can more directly capture the formation and expansion of localized high-temperature and high-stress areas (Pradawet et al., 2023; Zhang et al., 2023; Liao et al., 2024).

After tasseling, both distributional and mean features were selected, and mean features became more important for CWC estimation. Although dispersion features were still frequently selected by RFECV, their contribution to prediction accuracy was less consistent across models. This suggests that within-plot variability remained present after tasseling, but did not always improve model performance. A possible explanation is that reproductive growth, canopy closure, and senescence increased the influence of overall canopy status on CWC, making mean spectral and thermal features more representative of plot-level variation. Therefore, the contribution of statistical features should be considered stage- and model-dependent rather than universally superior to mean features.

It should be noted that, although the models captured the overall variation trend of CWC reasonably well, the observed-versus-predicted scatter plots still showed some compression in the responses to high- and low-value samples. This may be related to imbalanced sample distributions and the limited number of extreme samples (Ahmad et al., 2021). Recent studies have similarly shown that multimodal UAV data, thermal infrared–multispectral fusion, and model updating strategies can improve the stability of crop water-status estimation. However, under complex field conditions, underestimation of high-value samples or shrinkage in the prediction distribution may still occur (Liu et al., 2025; Yang et al., 2025).

### 4.2 Effects of simulated spatial aggregation on statistical feature responses

In this study, mean aggregation was used to simulate the response of spatial-resolution degradation under a consistent image-processing workflow. Therefore, the results should be interpreted as a post-processing-based scale-sensitivity analysis, rather than as direct evidence from real UAV observations acquired at different flight altitudes or with different native sensor resolutions. As the aggregation scale increased from 2 × 2 to 8 × 8, the correlation directions between representative high-frequency RFECV-selected features and CWC generally remained stable, whereas the correlation strength of some dispersion-related features weakened. This indicates that simulated aggregation mainly changed the strength of canopy-signal distributional information expressed in image features, rather than reversing the feature–CWC relationships. Previous multi-scale studies also suggest that local hotspots and small patches are more easily averaged out at coarser spatial resolutions, but direct transfer from simulated aggregation to real multi-altitude observations should be made cautiously (Sagan et al., 2019; Adedeji et al., 2025).

This scale response is closely related to the mathematical meaning of distributional statistical features. Standard deviation and interquartile range describe the width and dispersion of pixel-value distributions within plots, and are therefore sensitive to local extremes, boundaries, and patch structures. When neighboring pixels are averaged, localized high-temperature pixels, low-spectral-response pixels, and transition zones are smoothed, leading to narrower distributions and reduced variance. This is consistent with previous thermal studies showing that canopy-temperature variability can contain useful water-stress information, especially when high-resolution thermal imagery preserves local temperature differences (Han et al., 2016; González-Dugo et al., 2006). In contrast, mean features are already aggregated descriptors of overall canopy conditions and are therefore generally less sensitive to further spatial averaging (DeJonge et al., 2015).

The scale response also depended on growth stage and model type. Early water deficit may first appear as local changes in image features rather than as clear shifts in plot-level mean values (Zhuang et al., 2017; Asaari et al., 2019; Zhang et al., 2019b; Niu et al., 2021). The effect of simulated aggregation was not uniform across models, indicating that the usefulness of distributional features remained stage-, scale-, and model-dependent (Shao et al., 2023; Zhang et al., 2023; Liao et al., 2024).Therefore, from an application perspective, the appropriate spatial scale should be considered according to the monitoring objective, but this implication should be limited to the simulated aggregation framework used in this study. Preserving finer spatial information may help retain local distributional signals before tasseling, whereas moderate aggregation may improve the stability of mean canopy responses in some post-tasseling models. However, real changes in flight altitude or sensor resolution may also affect image sharpness, viewing geometry, shadow patterns, atmospheric path effects, bidirectional reflectance characteristics, and sensor noise. Therefore, the present results should be interpreted as evidence of scale sensitivity under controlled post-processing aggregation, and future studies using real multi-altitude, multi-platform, or UAV–satellite observations are needed to test their transferability (Wei et al., 2021; Liu et al., 2025; Yang et al., 2025).

### 4.3 Supplementary site-internal analysis at the Xinxiang site

In 2025, a summer maize irrigation experiment was conducted simultaneously in Xinxiang City, Henan Province. The field experiment followed a split-plot design with three replicates, in which irrigation treatment was the main-plot factor and maize cultivar was the subplot factor. The three irrigation treatments and four cultivars formed 12 irrigation–cultivar combinations, resulting in 36 plots in total. Each plot measured 5.2 m × 9 m. Three irrigation treatments, W3, W4, and W5, were established. UAV thermal infrared and multispectral imagery were acquired on July 26, August 11, August 31, and September 14, 2025, while LAI and EWT were also measured to calculate CWC. Compared with the Shiyanghe site, the Xinxiang site had fewer observation dates and a more limited irrigation-treatment gradient. Therefore, the Xinxiang dataset was used as a supplementary site-internal analysis scenario under weak water-gradient conditions. It should be emphasized that this analysis was not intended as a strict external validation or cross-site transfer test, because both model training and testing were conducted using samples from the Xinxiang site. The two sites had comparable experimental layout, but differed in climatic background: Shiyanghe was located in an arid oasis region with stronger evaporative demand, whereas Xinxiang was more affected by summer rainfall and humid conditions, which may have weakened the irrigation-induced water gradient and partly masked canopy heterogeneity signals.

**Figure 11:**
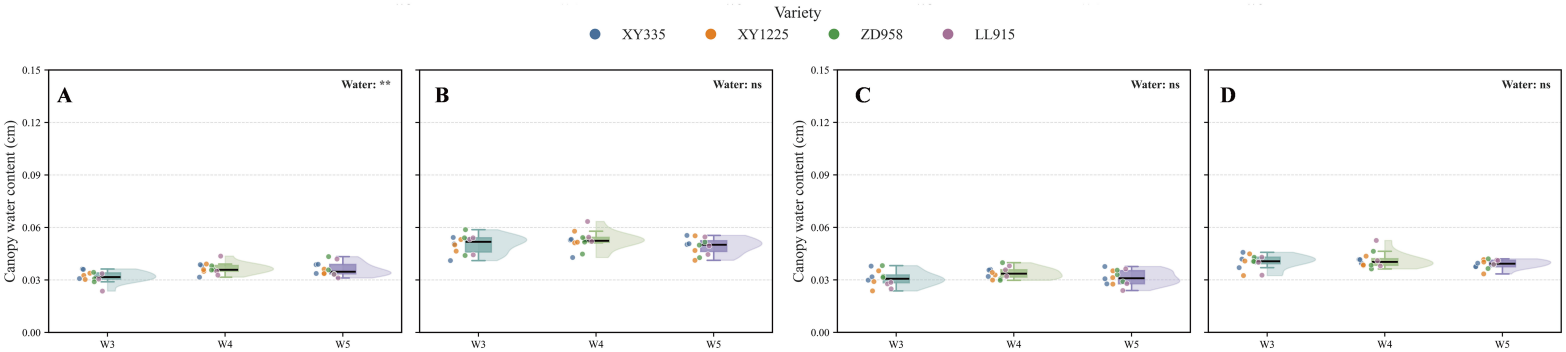
Changes in maize canopy water content under different growth stages and irrigation treatments at the Xinxiang site. **(A–D)** represent the jointing stage (2025-07-26), tasseling–silking stage (2025-08-11), grain-filling stage (2025-08-31), and maturity stage (2025-09-14), respectively. Asterisks indicate the overall significance of the main effect of irrigation treatment among W3, W4, and W5 within each growth stage, as determined by two-way analysis of variance. ns, *, **, and *** indicate p ≥ 0.05, p< 0.05, p< 0.01, and p< 0.001, respectively.

As shown in **Figure 11**, treatment separation in maize CWC at the Xinxiang site was generally weak and lacked persistence. A certain degree of differentiation in CWC distribution was observed only at the jointing stage, indicating a temporary water gradient during this period. However, at the tasseling–silking stage, grain-filling stage, and maturity stage, the overall main effect of irrigation treatment was not significant.

Given the limited number of observation dates and sample size at the Xinxiang site, further stage-specific modeling before and after tasseling would have resulted in insufficient training samples and reduced the stability of model evaluation. Therefore, site-internal modeling was conducted using samples from the whole growing season. Although whole-season pooling increased the sample size for model training, the limited number of observations still constrained the ability of machine-learning models to learn complex heterogeneity-related relationships. This is especially important for dispersion-based features, such as sd and iqr, because they depend strongly on extreme pixels or distribution tails; under weak-gradient conditions, insufficient extreme samples may make their relationships with plot-level CWC unstable. To improve the robustness of model evaluation, the Xinxiang analysis was performed using 50 repeated random train–test splits. In each repetition, RFECV feature selection was conducted only within the training set, and the same split was used to compare the mean-only and RFECV-selected feature sets. Model performance was summarized as mean ± standard deviation, and paired Wilcoxon tests were used to evaluate the significance of feature-set differences.

The RFECV selection-frequency analysis showed that canopy distributional information was not absent at the Xinxiang site. Several dispersion-related spectral features were frequently selected, including NDRE_iqr, GNDVI_iqr, and NDVI_iqr, with selection frequencies of 100%, 98%, and 94%, respectively. However, the predictive contribution of these features was model-dependent. The overall best-performing model was RFR with the mean-only feature set, with an R² of 0.508 ± 0.136 and an RMSE of 0.00564 ± 0.00077. Under the RFECV-selected feature set, the best-performing model was also RFR, with an R² of 0.504 ± 0.131 and an RMSE of 0.00568 ± 0.00077. The difference between the two RFR-based feature sets was not statistically significant. The Xinxiang results suggest that the contribution of RFECV-selected statistical features was scenario- and model-dependent, rather than universally beneficial. Under weak-gradient and small-sample conditions, RFECV-selected features may capture useful local distributional information, but this information may not consistently translate into improved overall prediction performance. **Supplementary Figure 1** shows the repeated-split site-internal plot-level spatial distribution of predicted CWC at the Xinxiang site.

### 4.4 Limitations and future perspectives

Although this study explored UAV-based maize CWC estimation from the perspectives of canopy heterogeneity and spatial scale responses, several limitations remain. The main modeling and scale-effect analyses were based on single-year experimental data from the Shiyanghe site. Although different irrigation treatments and cultivar combinations were included, interannual variations in meteorological conditions, soil background, management practices, and crop growth processes may alter canopy water status and its remote sensing responses. Therefore, the stability of statistical features for CWC estimation across different years, climatic backgrounds, and cropping systems still requires further validation. Future studies should incorporate multi-year and multi-site experimental data to further evaluate the general applicability of statistical features for CWC estimation and to clarify the water-gradient ranges and growth-stage conditions under which they are most effective. It should also be noted that the ground-measured CWC in this study was derived from plot-level LAI and EWT observations. Therefore, the model outputs represent plot-level CWC estimates rather than pixel-level CWC distributions. The canopy heterogeneity emphasized in this study refers to complementary predictive information contained in within-plot pixel-value distributions from high-resolution UAV imagery. Future studies aiming to map continuous within-canopy CWC variation will require ground validation data with higher spatial correspondence or a local-window-based validation framework.

Although the 50 repeated random splits reduced the dependence of model evaluation on a single sample partition, this strategy cannot fully remove potential dependence among samples from the same observation date, cultivar, irrigation treatment, or plot. Therefore, future studies with larger multi-year and multi-site datasets should further evaluate model generalizability using stricter grouped validation or independent external validation.

The Xinxiang site was used mainly as a supplementary validation scenario under weak water-gradient conditions, based on site-internal training and testing rather than strict cross-site external validation. Because the Xinxiang site had fewer observation dates and lacked persistent treatment separation in CWC, the gain from RFECV-selected features was not fully expressed. This result suggests that the effectiveness of canopy-signal distributional features may depend on whether water stress can form a stable spatial expression pattern. Future studies should conduct external validation at more sites with clear water gradients and continuous observation dates to further test the cross-regional transferability of statistical features and the proposed modeling framework.

In addition, this study used mean aggregation to simulate spatial resolution degradation and focused on changes in statistical features and model performance at the 2 × 2, 4 × 4, and 8 × 8 aggregation scales. This approach can reflect the smoothing effect of spatial aggregation on pixel-distribution information, but it does not fully account for the effects of different sensor imaging mechanisms, flight-altitude changes, and image-registration errors on scale effects. Future work should combine images acquired at different actual flight heights, multi-platform imagery, or UAV–satellite multisource data to further distinguish between simulated scale changes and real observation-scale changes. This would provide a stronger basis for transferring CWC estimation models across different remote sensing platforms.

## 5 Conclusion

This study used UAV-based multispectral and thermal infrared imagery to evaluate whether image-derived canopy-signal distributional features could provide complementary information for plot-level maize CWC estimation. Mean, percentile, and dispersion features were extracted from effective canopy pixels within each plot, and their contributions were examined across growth stages and simulated spatial aggregation scales. Model performance was evaluated using repeated random train–test splits and paired statistical tests.

At the Shiyanghe site, water deficit was associated with changes in both overall canopy spectral–thermal responses and within-plot canopy-signal distributions. The expansion of high-CWSI anomalous areas and larger connected patches indicated that water-stress responses were not always expressed as uniform canopy-wide changes, but could also appear as localized and patch-like thermal patterns. These results support the use of pixel-distribution-based features as complementary descriptors of plot-level canopy water status, while recognizing that these features may also be affected by canopy structure and image-related factors.

The contribution of statistical features to CWC estimation was growth-stage and model dependent. Before tasseling, RFECV selection was more frequently associated with percentile and dispersion features, suggesting that lower-tail spectral responses and within-plot signal variability contained useful CWC-related information. However, the improvement provided by the RFECV-selected feature set was not universal across models. After tasseling, mean features generally showed more stable performance in several models, although some distributional features still contained CWC-related information. The supplementary Xinxiang site-internal analysis further indicated that, under weak water-gradient and small-sample conditions, distributional features may be frequently selected but may not consistently improve prediction accuracy.

Simulated spatial aggregation affected the strength of feature–CWC relationships and the predictive contribution of distributional features. As the aggregation scale increased from 2 × 2 to 8 × 8, the correlation directions of representative RFECV-selected features generally remained stable, but the correlations of several dispersion-related features weakened. Under the simulated aggregation framework used in this study, finer spatial information was more useful for retaining local distributional signals before tasseling, whereas mean features were more stable in several post-tasseling models. Overall, the results suggest that image-derived canopy-signal distributional features can provide useful complementary information for plot-level maize CWC estimation, but their value is stage-, scale-, and model-dependent. Future studies using multi-year, multi-site, and real multi-altitude or multi-platform observations are needed to further test the transferability of these findings.

## Data Availability

The data analyzed in this study is subject to the following licenses/restrictions: The data are not publicly available due to confidentiality requirements but may be made available from the corresponding author upon reasonable request and with permission. Requests to access these datasets should be directed to Haiyang Zhang, zhy_cau@cau.edu.cn.

## Supplementary material

Supplementary Figure 1 Cross-validated plot-level spatial distribution of predicted CWC at the Xinxiang site across four flight dates. For each plot-date observation, the mapped value represents the mean test-set prediction obtained across the 50 repeated random splits. **(A)** 2025-07-26; **(B)** 2025-08-11; **(C)** 2025-08-31; **(D)** 2025-09-14. W3–W5 indicate irrigation treatments. These maps represent plot-level CWC predictions rather than pixel-scale CWC retrievals.
