# Supplementary figures and images for: Estimating maize canopy water content using UAV-based multispectral–thermal infrared imagery and canopy signal distributional features
Source: Front Plant Sci. 2026 Jul 9;17:1868370. doi: 10.3389/fpls.2026.1868370 (PMC13391866; doi:10.3389/fpls.2026.1868370)

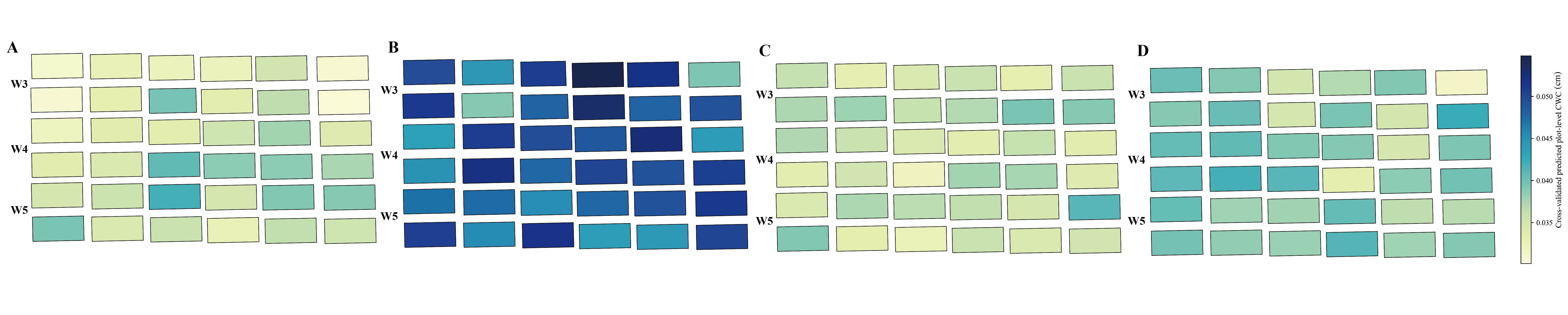

Supplement: Supplementary Figure 1 — Cross-validated plot-level spatial distribution of predicted CWC at the Xinxiang site across four flight dates. For each plot-date observation, the mapped value represents the mean test-set prediction obtained across the 50 repeated random splits. (A) 2025-07-26; (B) 2025-08-11; (C) 2025-08-31; (D) 2025-09-14. W3–W5 indicate irrigation treatments. These maps represent plot-level CWC predictions rather than pixel-scale CWC retrievals. [file Image1.jpeg]
