# Supplementary material for: Estimating maize canopy water content using UAV-based multispectral–thermal infrared imagery and canopy signal distributional features
Source: Front Plant Sci. 2026 Jul 9;17:1868370. doi: 10.3389/fpls.2026.1868370 (PMC13391866; doi:10.3389/fpls.2026.1868370)
Supplement: Supplementary file 2 [file Table1.docx]

Supplementary Table S1. Summary of observation dates and final plot-level CWC samples used for model development.

| Site | Growth stage | Water treatment | Cultivar | Experimental design | Observation dates in 2025 | Number of plots | Plot-level CWC samples | Final samples used for |
| --- | --- | --- | --- | --- | --- | --- | --- | --- |
| Shiyanghe | Pre-tasseling | W1-W5 | XY335, XY1225, ZD958 | Split-plot; irrigation as main plot, cultivar as subplot | June 6, June 20, June 30 | 45 per date | 3×45=135 | 135 |
| Shiyanghe | Post-tasseling | W1-W5 | XY335, XY1225, ZD958 | Split-plot; irrigation as main plot, cultivar as subplot | July 15, July 22, August 15, August 23 and September 13 | 45 per date | 5×45=225 | 225 |
| Xinxiang | Full growth stage | W3-W5 | XY335, XY1225, ZD958, Li Long915 | Split-plot; irrigation as main plot, cultivar as subplot | July 26, August 11, August 31, and September 14 | 36 per date | 4×36=144 | 144 |

Note: Plot-level CWC samples refer to the number of valid plot-level ground measurements obtained after averaging LAI and EWT measurements within each plot.

Supplementary Table S2. Date-specific Otsu NDVI thresholds and canopy-mask keep ratios used for vegetation masking.

| Site | Flight date | Growth stage | Otsu NDVI threshold | Mask keep ratio |
| --- | --- | --- | --- | --- |
| Shiyanghe | June 6 | Pre-tasseling | 0.3398 | 0.3440 |
|  | June 20 |  | 0.4570 | 0.5626 |
|  | June 30 |  | 0.5742 | 0.7272 |
|  | July 15 | Post-tasseling | 0.5508 | 0.7103 |
|  | July 22 |  | 0.5742 | 0.7346 |
|  | August 15 |  | 0.5039 | 0.7222 |
|  | August 23 |  | 0.5820 | 0.8256 |
|  | September 13 |  | 0.4805 | 0.5719 |
| Xinxiang | July 26 | Full growth stage | 0.6055 | 0.7404 |
|  | August 11 |  | 0.6368 | 0.8318 |
|  | August 31 |  | 0.6680 | 0.8519 |
|  | September 14 |  | 0.6523 | 0.8498 |

Note: The NDVI threshold was determined separately for each flight date using Otsu’s method. Mask keep ratio refers to the proportion of pixels retained after NDVI thresholding and morphological filtering.

Supplementary Table S3. Main model settings and hyperparameter search ranges used for CWC estimation.

| Model | Tuned hyperparameters | Candidate values or search ranges |
| --- | --- | --- |
| GBDT | n_estimators; learning_rate; max_depth; subsample; min_samples_split; min_samples_leaf; max_features | n_estimators = [100, 300, 500, 800]; learning_rate = [0.005, 0.01, 0.02, 0.05, 0.1]; max_depth = [2, 3, 4, 5]; subsample = [0.5, 0.6, 0.8, 1.0]; min_samples_split = [2, 4, 6, 8]; min_samples_leaf = [1, 2, 3, 4]; max_features = [None, sqrt, log2, 0.5, 0.8] |
| RFR | n_estimators; max_depth; min_samples_split; min_samples_leaf; max_features;bootstrap | n_estimators = [300, 500, 800, 1200]; max_depth = [None, 6, 8, 10, 12, 15]; min_samples_split = [2, 3, 5, 8]; min_samples_leaf = [1, 2, 3, 4]; max_features = [sqrt, log2, 0.4, 0.6, 0.8, 1.0]; bootstrap = [True, False] |
| PLSR | n_components | n_components = 1 to min (n_features, n_train − 1, 15) |
| SVR | C; gamma; epsilon | kernel = RBF; C = [0.1, 0.3, 1, 3, 10, 30, 100, 300]; gamma = [scale, auto, 0.0001, 0.001, 0.01, 0.1]; epsilon = [0.001, 0.01, 0.05, 0.1, 0.2] |

Note: The table summarizes the parameter search ranges and optimization settings used in the model-tuning procedure. Hyperparameter tuning was conducted only within the training set, and the test set was used only for final model evaluation.
